# Supplementary material for: Effects of emissions caps on the costs and feasibility of low-carbon hydrogen in the European ammonia industry
Source: Nat Commun. 2024 May 4;15:3753. doi: 10.1038/s41467-024-48145-z (PMC11069508; doi:10.1038/s41467-024-48145-z)
Supplement: Supplementary file 1 — Supplementary Information [file 41467_2024_48145_MOESM1_ESM.pdf]

Supplementary Information

Effects of emissions caps on the costs and feasibility of low-carbon hydrogen in the European ammonia industry

Stefano Mingolla[1]*, Paolo Gabrielli[2,3†], Alessandro Manzotti[4,5†], Matthew J. Robson[4†], Kevin Rouwenhorst[6,7,8], Francesco Ciucci[4,9], Giovanni Sansavini[2], Magdalena M. Klemun[10,11]*, Zhongming Lu[1,10]*

[1]Division of Environment and Sustainability, Hong Kong University of Science and Technology, Clear Water Bay, Kowloon, Hong Kong SAR, China
[2]Institute of Energy and Process Engineering, ETH Zurich, 8092, Zurich, Switzerland
[3]Department of Global Ecology, Carnegie Institution for Science, Stanford, CA 94305, USA
[4]Department of Mechanical and Aerospace Engineering, Hong Kong University of Science and Technology, Clear Water Bay, Kowloon, Hong Kong SAR, China
[5]Department of Physics, Technical University of Denmark, Kongens Lyngby, Denmark
[6]Ammonia Energy Association, 77 Sands Street, Brooklyn, NY 11201, USA
[7]Catalytic Processes & Materials, MESA+ Institute for Nanotechnology, Department of Science & Technology, University of Twente, P.O. Box 217, 7500 Enschede, The Netherlands
[8]Koolen Industries, Europalaan 202, 7553 SC Hengelo, The Netherlands
[9]Chair of Electrode Design for Electrochemical Energy Systems, University of Bayreuth, 95448 Bayreuth, Germany
[10]Energy Institute, The Hong Kong University of Science and Technology, Hong Kong SAR, China
[11]Division of Public Policy, Hong Kong University of Science and Technology, Clear Water Bay, Kowloon, Hong Kong SAR, China

↵* Corresponding authors. Email: smingolla@connect.ust.hk, zhongminglu@ust.hk, magdalena@ust.hk
↵† These authors contributed equally to this work and are listed in alphabetic order.

Supplementary Note 1

In the "Fit for 55 package", the European Union has updated the Renewable Energy Directive II with a delegated act[1]. This act outlines requirements for additionality and temporal and geographic correlation for electricity used in hydrogen and derived fuels production. This ensures that increased electricity demand for hydrogen production does not lead to a rise in fossil fuel-based electricity generation, nor does it compromise the electricity system's operational efficiency. The additionality principle emphasizes the need for hydrogen production to be powered by new electricity generation capacity rather than relying on existing renewable sources. Temporal correlation seeks to align the timing of electricity use for hydrogen production with periods of renewable electricity generation. Lastly, renewable electricity sources and hydrogen production plants should be located near each other, thereby minimizing transmission distances and potential grid congestion[2,3].

Supplementary Note 2

There is no unique dataset listing the European ammonia plants. The main ammonia producers in Europe are Yara, Fertiberia, BASF, and Borealis. From their websites, the main production sites in Europe were located. Furthermore, production sites were compared with the list provided by the European Commission[4] and the association Fertilizer Europe[5]. Lastly, since not all ammonia producers indicate in which ammonia plant hydrogen is synthesized, locations were also compared with the list of the major steam methane reforming (SMR) hydrogen generation units provided by the (Fuel Cell Hydrogen Observatory) FCHO[6]. Therefore, it is possible to affirm that most of the current major European ammonia plants are included in the dataset, which gives a realistic image of the industry. Once more updated datasets are available, eventual missing ammonia plants can be easily included in the model.

Supplementary Note 3

The carbon dioxide equivalent (CO_2e) emissions associated with manufacturing utility-scale photovoltaic (PV) panels and wind turbines have been collected from Nugent and Sovacool[7]. The authors report an average emission intensity of 50 kilograms (kg) CO_2e per megawatt hour (MWh) for solar PV and 35 kg CO_2e/MWh for wind energy. By applying global average capacity factors, the emission content per MW installed capacity is 787 tons CO_2e/MW for solar PV and 1,345 tons CO_2e/MW for wind turbines. Similarly, data from Gerloff indicates that the emission content for large-scale electrolyzers is 133 tons CO_2e/MW installed[8]. Furthermore, Palmer et al. provide the emissions from large-scale hydrogen storage tanks, which amount to 0.182 tons CO_2e per kilogram of hydrogen capacity[9]. This emission factor is also applicable to large-scale hydrogen compressors when measured per kilogram of hydrogen compressed per hour.

Supplementary Note 4

The installation cost I_k is equal to the cost of the technology c_k multiplied by the installed capacity $\dot{P}_k$ (equation 1).

$$I_k = c_k \left(\dot{P}_k \right) \tag{1}$$

Operation and maintenance (O&M) v_k are presented as a fraction of the installation cost.

Supplementary Note 5

PV and WT installations

The hourly power generation, $V_{\mathrm{E},k_R,t}$ from photovoltaics (PV) and wind turbines (WT) results from (equation 2).

$$V_{\mathrm{E},k_R,t}=\eta_{k_R}\left(\omega_{R,t}\right)\dot{P}_{k_R},\ \forall\ k\in K_R \quad (2)$$

Where K_R is the set of renewable energy technologies {PV, WT}; η_{k_R} is the transmission efficiency of solar PV and WT; $\omega_{R,t}$ is the time-dependent capacity factor of solar and wind energy ($R\in\{\text{solar, wind}\}$) and $\dot{P}_{k_R}$ is the installed capacity of PV and WT.

Li-ion batteries

The amount of renewable electricity stored in a Battery Energy Storage System (BESS) at a given hour t, denoted as $S_{\mathrm{E,B},t}$, is determined by the following process:

Firstly, the energy remaining from the previous hour $S_{\mathrm{E,B},t-1}$ is reduced by the energy lost through the batteries' self-discharge over time, quantified as the self-discharge rate $\lambda_{\mathrm{B}}\Delta_t$. To this adjusted value, the amount of electricity fed into the BESS during hour t $U_{\mathrm{E,B},t}$ is added, with the quantity modified by the battery's charging efficiency $\eta_{\mathrm{B}}^{\mathrm{c}}$. Finally, the energy dispensed from the BESS during the same hour $V_{\mathrm{E,B},t}$ is subtracted, which is scaled by the discharging efficiency $\eta_{\mathrm{B}}^{\mathrm{d}}$ (equation 3).

$$S_{\mathrm{E,B},t}=(1-\lambda_{\mathrm{B}}\Delta_t)S_{\mathrm{E,B},t-1}+\eta_{\mathrm{B}}^{\mathrm{c}}U_{\mathrm{E,B},t}-\frac{V_{\mathrm{E,B},t}}{\eta_{\mathrm{B}}^{\mathrm{d}}},\ \forall\ t\in\{1,\ ...,\ T\} \quad (3)$$

The amount of electricity entering the BESS and the hourly maximum output of the BESS are constrained by the minimum number of time intervals for full charge, τ^{c} and discharge, τ^{d} (equation 4) and (equation 5) while the quantity stored at time t is constrained by the maximum installed capacity $\dot{P}_{\mathrm{B}}$ (equation 6).

$$0\leq U_{\mathrm{E,B},t}\leq\frac{\dot{P}_{\mathrm{B}}}{\tau^{\mathrm{c}}},\ \forall\ t\in\{0,\ ...,\ T\} \quad (4)$$

$$0 \leq V_{E,B,t} \leq \frac{\dot{P}_B}{\tau^d},\ \forall\ t \in \{0,\ ...,\ T\} \tag{5}$$

$$0 \leq S_{E,B,t} \leq b_B \dot{P}_B,\ \forall\ t \in \{0,\ ...,\ T\} \tag{6}$$

Electrolyzers

We set a minimum constraint for the installed capacity of the electrolyzer, denoted as $\dot{P}_{EL,min}$. This minimum capacity is established to ensure a 100% capacity factor, indicating that the electrolyzer operates continuously without any periods of downtime (equation 7).

$$\dot{P}_{EL,min} \leq \dot{P}_{EL} \leq \dot{P}_{EL,max} \tag{7}$$

$\dot{P}_{EL,min}$ results from (equation 8), where $\eta_{H2,E,EL}$ the electrolyzer conversion efficiency.

$$\dot{P}_{EL,min} = \rho\left(D^{h}_{NH3}\right)\eta_{H2,E,EL} \tag{8}$$

The model optimizes the hourly hydrogen output of the electrolyzer, $V_{H2,EL,t}$ by considering the conversion efficiency of the electrolyzer, $\eta_{E,H2,EL}$, which is multiplied by the input electricity during the corresponding hour, $U_{E,EL,t}$, according to (equation 9).

$$V_{H2,EL,t} = \eta_{E,H2,EL}\left(U_{E,EL,t}\right),\ \forall\ t \in \{0,\ ...,\ T\} \tag{9}$$

Hydrogen compressor and high-pressure storage tanks

The model optimizes the hourly input electricity, $U_{E,CP,t}$ and output hydrogen of the compressor, $V_{H2,CP,t}$ as follow (equation 10):

$$V_{H2,CP,t} = \eta_{E,H2,CP}\left(U_{E,CP,t}\right),\ \forall\ t \in \{0,\ ...,\ T\} \tag{10}$$

Where the conversion efficiency of the electrolyzer is $\eta_{E,H2,CP}$. Notably, we assume no hydrogen losses during the compressor phase (equation 11).

$$U_{H2,CP,t}=V_{H2,CP,t}, \forall\ t \in \{0, \ldots, T\} \tag{11}$$

Once compressed, the output hydrogen of the compressor, $V_{H2,CP,t}$, directly becomes the input hydrogen of the storage tanks, $U_{H2,ST,t}$ (equation 12).

$$U_{H2,ST,t}=V_{H2,CP,t}, \forall\ t \in \{0, \ldots, T\} \tag{12}$$

At each time step t, the amount of stored hydrogen (in kilograms), $S_{H2,ST,t}$ is determined by adding the previously stored hydrogen, $S_{H2,ST,t-1}$, to the incoming hydrogen from the compressor, $U_{H2,ST,t}$, and subtracting the hydrogen that exits the storage tanks, $V_{H2,ST,t}$ for use in the ammonia synloop (equation 13). No self-discharge losses are considered for hydrogen storage[10,11].

$$S_{H2,ST,t}=S_{H2,ST,t-1}+U_{H2,ST,t}-V_{H2,ST,t}, \forall\ t \in \{1, \ldots, T\} \tag{13}$$

Supplementary Note 6

Local algorithms were tested first to set a baseline and subsequently search for other extrema with more explorative global algorithms. In some cases, for narrow or less complex problems, local algorithms may outperform global one[12]. These last are well-developed algorithms that are preferable in most of the problems[13,14].

The first local algorithm selected was Nelder-Mead. This is a standard and widely used direct algorithm for a multi-dimensional nonlinear minimization problem. It is also defined as a "pattern search optimization algorithm" where the function's gradient is not known[15–17]. Is it necessary to provide a starting point for the algorithm that can be a random point from the domain region or the results of previous optimization if a hybrid approach is chosen[15]. The selected initial coordinates were verisimilar estimates based on pre-existing knowledge about the hydrogen energy system. Multiple trials with those coordinates led to the same results (difference $<10^{-8}$ among tests), showing that the result can be considered a global optimum candidate. However, when repeating the test with random coordinates within the bounds, the results were less accurate and subject to higher variability (Supplementary Table 12). It is possible to conclude that although Nelder-Mead is a good candidate algorithm, prior knowledge of the approximate expected solution is needed to approach the optimum.

The second test was conducted with L-BFGS-B, one of the most used algorithms for numerical optimization. L-BFGS-B is a limited-memory quasi-Newton second-order local algorithm for solving large nonlinear optimization problems[12,14,17]. The same tests conducted for Nelder-Mead (same initial coordinates and set of regions) were performed for L-BFGS-B. Although Nelder-Mead and L-BFGS-B are different algorithms belonging to different sub-categories of algorithms (the first is a "direct algorithm" while the second is a "second-order algorithm"), the results were similar (in most cases <0.01 % differences) and consistent across the tests conducted. Similarly to Nelder-Mead, random initial coordinates tend to result in lower accuracy (higher LCOH) (Supplementary Table 12).

Finally, global algorithms were tested, starting with population algorithms. These are nature-inspired "population-based metaheuristic search algorithms" that iteratively improve candidate solutions on an evolutionary process to optimize an optimization problem[18]. Population algorithms such as genetic algorithms (GA) and differential evolution (DE) are generally used for global search. Both are usually implemented in case of more challenging multimodal and noisy functions where other methods cannot find a good solution[12,14,19]. However, one of the drawbacks is the high computational cost compared to the previously described local methods.

DE was the global search algorithm tested. Like genetic and other evolutionary algorithms, DE starts with an initial population of candidate solutions. Then, these are iteratively improved by introducing

mutations into the population, retaining the fittest candidate solutions (the one with lower objective function value, in this case, LCOH)[19]. Compared to other evolutionary algorithms, the benefit of using DE is that can work with nonlinear and non-differentiable multi-dimensional objective functions while requiring very few control parameters[17,19]. In addition, it is easy and practical to use and presents good convergence properties (consistent convergence to the global minimum in consecutive independent trials[19]).

In DE, three key hyperparameters can be tuned to improve the chances of finding a global minimum: (i) population size, (ii) mutation (dithering), (iii) recombination values[20]. This widens the search radius but slows the convergence of the algorithm. The DE algorithm was initialized with the default hyperparameters, and several optimizations were performed for each region with consistent results. Those results were in line with the ones of local algorithms, although slightly less accurate in most cases (Supplementary Table 8). Subsequently, the effect of a change in population size was studied, testing how higher and lower populations would perform and how long the algorithm took to converge. Results show no significant benefit in terms of higher accuracy when increasing population size.

From the tests conducted, the following was observed: (i) both local and global algorithms converged to the some LCOH (< 0.1% difference) although the coordinates may differ by higher percentages; (ii) higher population size significantly increased computational time but did not directly translate in higher accuracy (lower LCOH); (iii) when re-testing the same DE's hyperparameters consistent results were obtained, and (iv) local algorithms performed better only when verisimilar initial coordinates were given (Supplementary Table 12). Since both local and global approaches resulted in approximately the same LCOH, it was possible to affirm that the results approach global minima for all the regions under assessment.

Supplementary Note 7

The electrolyzer system can be divided into two main components: stack and balance of plant (BoP). The stack is the core of the electrolyzer system, where electrochemical reactions occur. BoP comprises several subsystems (e.g., power supply, cooling pumps, oxygen separator tanks, rectifiers, etc.). While it is expected a relatively small reduction in the unit cost of the BoP (most of the components are already widely used in other applications), a significant and major reduction in the stack component is expected with economies of scale and major technological improvements[21]. The rate at which the unit cost of the stack component will decrease also depends on the type of electrolyzer.

Supplementary Note 8

Concerning the modifications necessary for transitioning an ammonia plant to electrolytic hydrogen production, it is necessary to replace the existing start-up heater with a new electric model ahead of the ammonia converter. Additionally, to accommodate flexible operation, supplementary electric heaters must be installed. In case of low-load operation, such as when the ammonia converter produces an inadequate amount of steam, steam generators will be required. The financial implication of these upgrades is estimated to be around 5-10% of the expense associated with constructing a new ammonia loop, equating to roughly 20 million euro (EUR)[22].

The operational lifespan of a steam methane reforming (SMR) system typically ranges from 25 to 30 years. However, the average ammonia plant has been operational for 15 years on average[23]. Consequently, when considering replacing SMR with an electrolytic hydrogen production system (EHPS), it is necessary to factor in the sunk costs associated with the early decommissioning of the SMR system. The initial cost of an SMR infrastructure is around 50 million EUR[24]. If decommissioned at the halfway mark of its lifespan, the undepreciated value—which represents the sunk cost—would be approximately 25 million EUR. This figure can be partially mitigated by the potential scrap value, which is estimated to be 10% of the original cost, equating to 5 million EUR. Therefore, after accounting for the scrap value, the net sunk cost incurred would amount to 20 million EUR[24].

Supplementary Note 9

$$P_t = P_{t-1} * \Delta P \quad (14)$$

where P_t = Price electricity at time t; P_{t-1}= Price electricity at time t-1; ΔP = Stochastic process representing the change in electricity price. It is the geometric Brownian motion (GBM) with drift parameter μ and volatility parameter σ.

The equation follows:

$$\Delta P = \exp\left[\left(\mu - \frac{\sigma^2}{2}\right)t + \sigma Z_t\right] \quad (15)$$

where Z_t = is a standard Brownian motion. The GBM model assumes a normal distribution of logarithmic returns and constant volatility, which simplifies the complexities of real-world financial markets where actual price returns may not follow a normal distribution and volatility can be dynamic. However, the robustness analysis and inclusion of pessimistic and optimistic scenarios compensate for these simplifications by evaluating a wide array of electricity price data across different regions. This approach has confirmed that the key conclusions of the study are consistent, even when considering variations in electricity costs and their potential volatility.

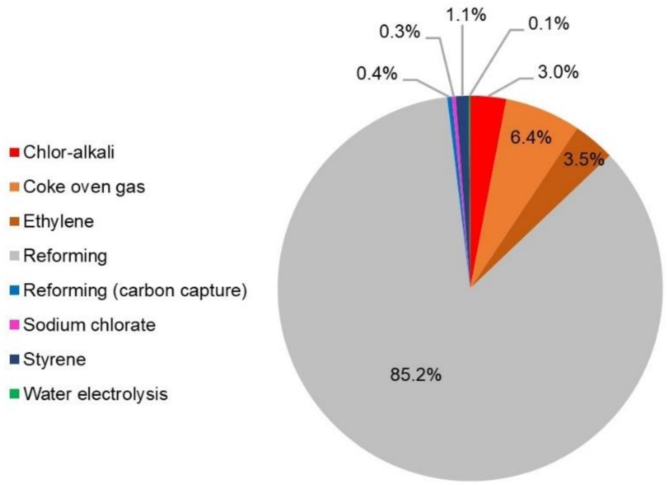

Supplementary Figure 1 | Hydrogen supply capacity by process (%). Authors' elaboration based on data taken from[6]. Steam methane reforming (SMR) is a mature production process that uses methane (CH_4) to produce hydrogen and is currently the most diffused, especially in Europe. The reaction of natural gas and high-temperature steam produces carbon monoxide and hydrogen, while a second reaction—water gas shift (WGS)— produces additional hydrogen and carbon dioxide.

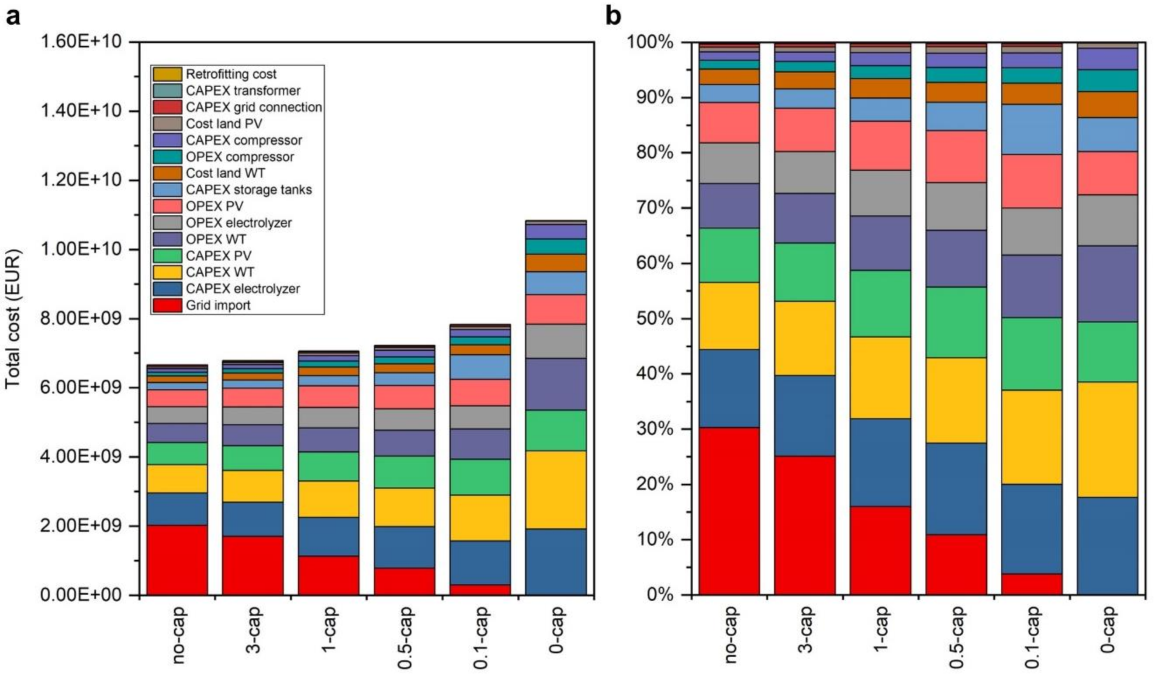

Supplementary Figure 2 | Mean electrolytic hydrogen production system (EHPS) total investment cost (capital expenditures (CAPEX) and operating expenditures (OPEX)) in euro (EUR) across European ammonia plants and under different emission caps. a total cost in EUR. **b** breakdown of relative cost contributions by cost item. The primary cost drivers are grid electricity import, CAPEX for the electrolyzer, and CAPEX for wind turbines and photovoltaic systems.

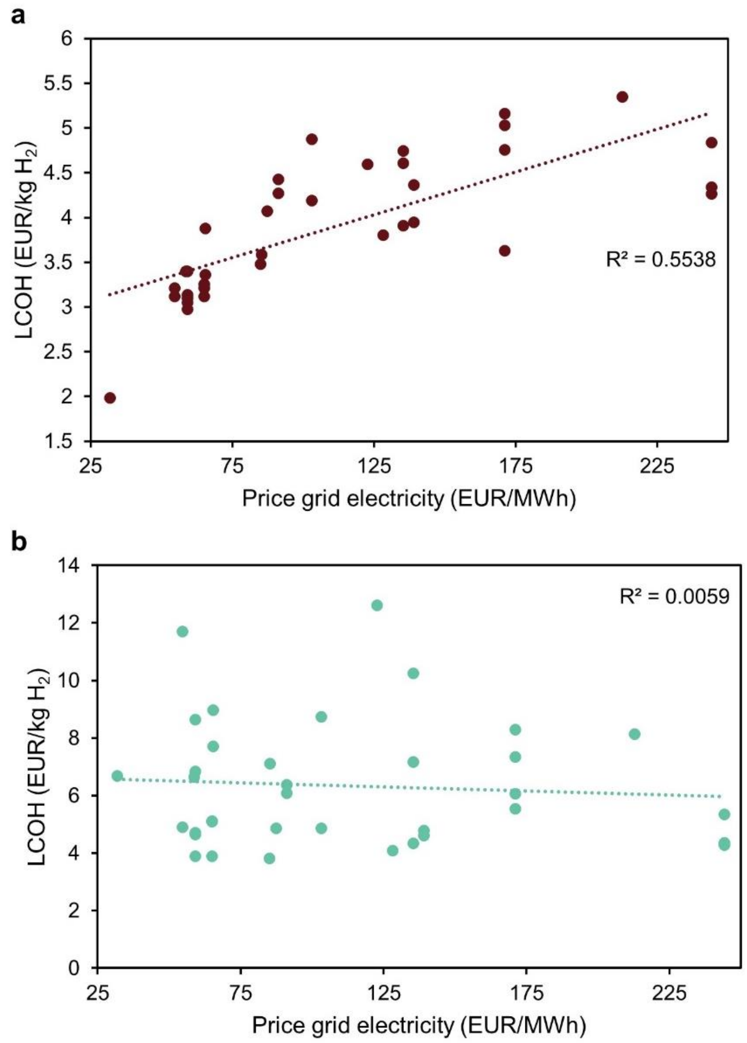

Supplementary Figure 3 | Correlation between the price of imported grid electricity in euro per megawatt hour (EUR/MWh) and the levelized cost of hydrogen (LCOH) in euro per kilogram of hydrogen (EUR/kg H_2) across European plants. a no-cap. **b** 0.1-cap. The correlation between grid electricity price and LCOH demonstrates a positive trend (coefficient of determination or R^2 = 0.55) under less stringent emission caps, which diminishes with stricter caps, leading to no significant correlation (R^2 = 0.01) under the 0.1 emission cap.

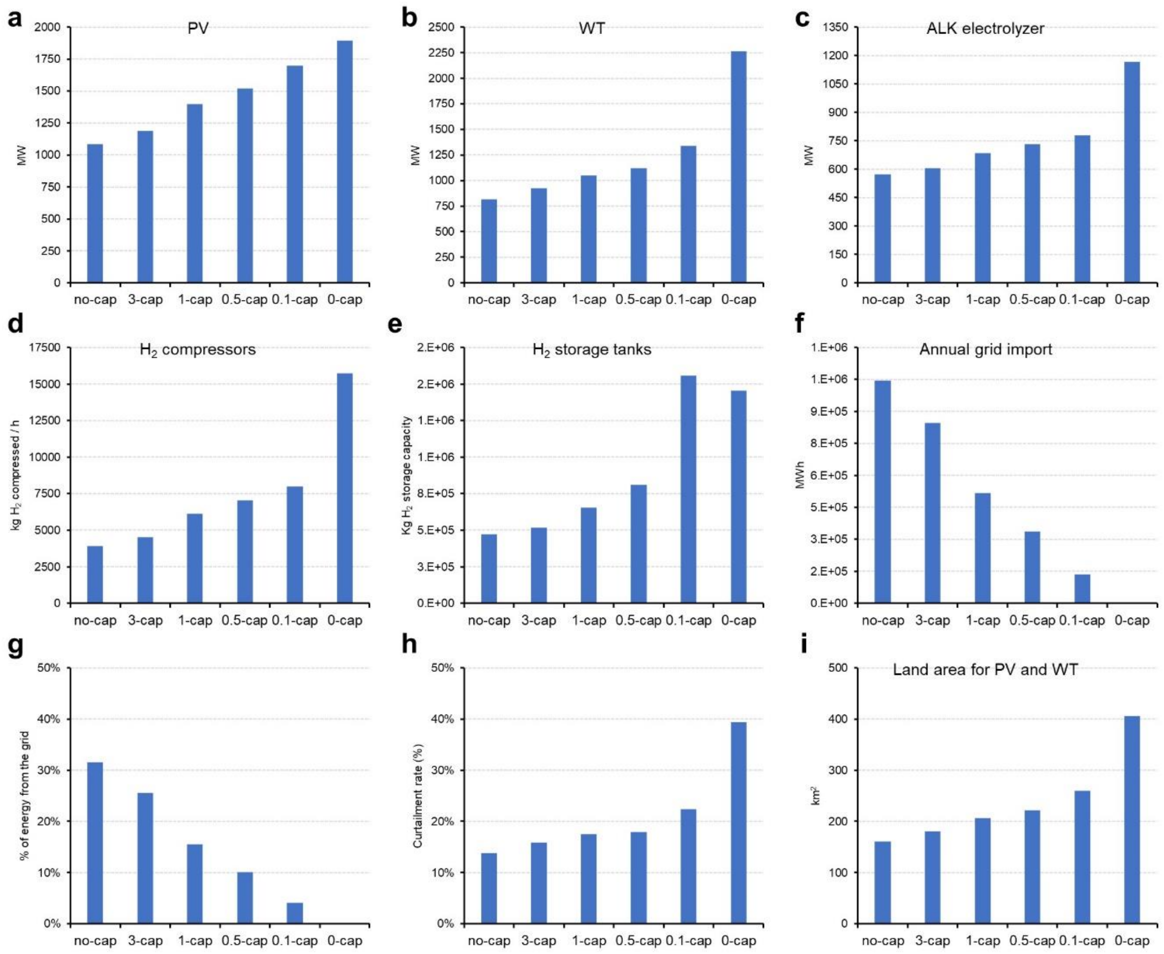

Supplementary Figure 4 | Average optimal electrolytic hydrogen production system (EHPS) design and operation for European plants. a installed capacity photovoltaics (PV) in MW. **b** installed capacity wind turbine (WT) in megawatt (MW). **c** installed capacity Alkaline (ALK) electrolyzers in MW. **d** installed capacity hydrogen compressors in kilogram of hydrogen compressed per hour (kg H_2 compressed/h). **e** installed capacity high-pressure hydrogen storage tanks in kg H_2 storage capacity. **f** annual grid import in megawatt hours (MWh). **g** grid-imported to renewable energy ratio. **h** renewable curtailment rate. **i** total land use for renewable installations in square kilometers (km^2).

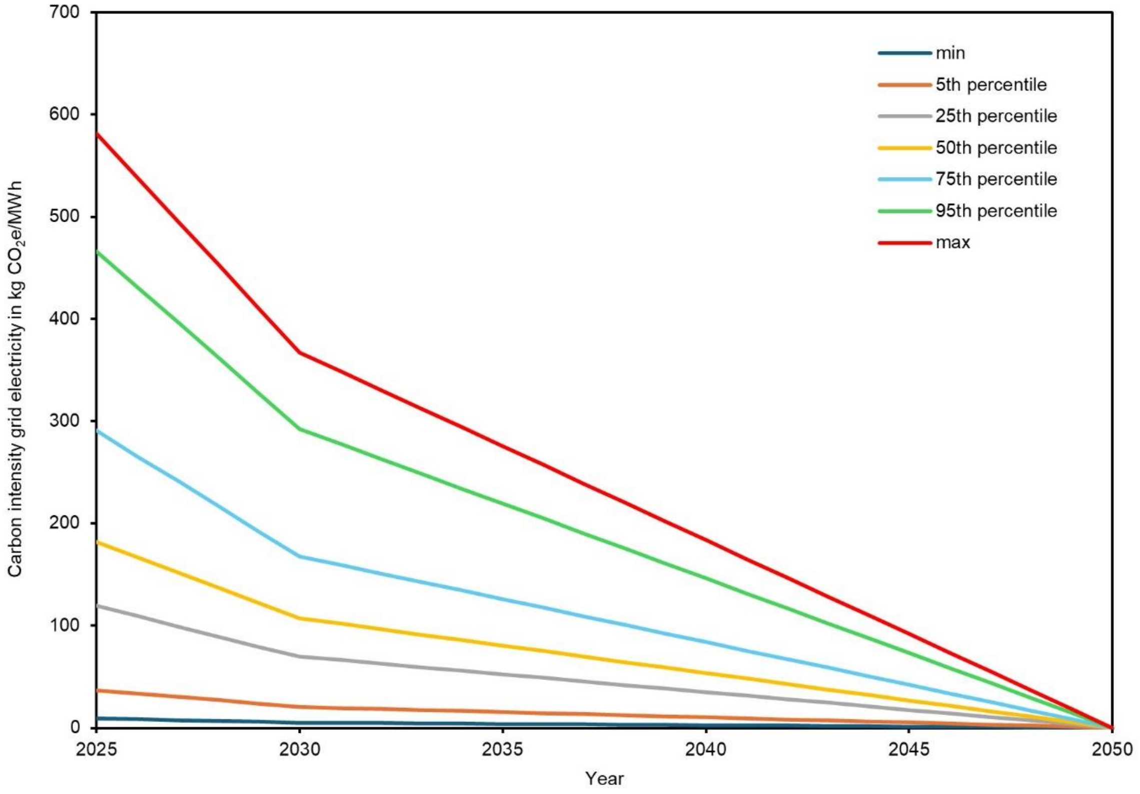

Supplementary Figure 5 | National carbon emission reduction. Trend in the carbon intensity of grid electricity used in this work based on JRC-COM-NEEFE (National and European Emission Factors for Electricity Consumption) historical data[25] and the European Environment Agency (EEA) projections[26]. Results under the no-cap and the reference case.

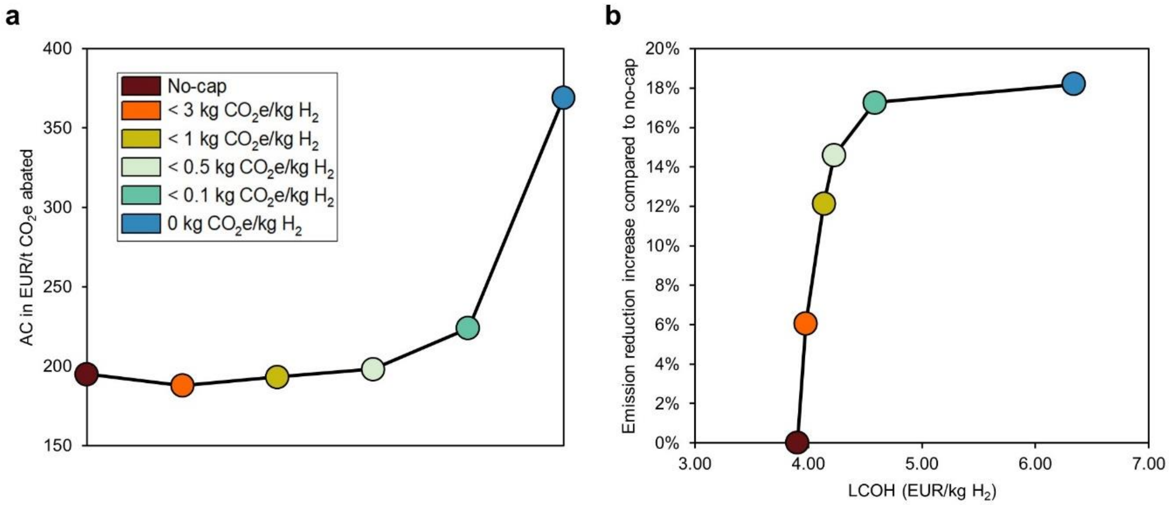

Supplementary Figure 6 | Average abatement cost (AC) across Europe – reference case. a AC in euro per tons of carbon dioxide equivalent (EUR/t CO_2e) abated. **b** levelized cost of hydrogen (LCOH) in euro per kilogram of hydrogen (EUR/kg H_2) (x-axis) vs. emission reduction increase compared to no-cap (y-axis).

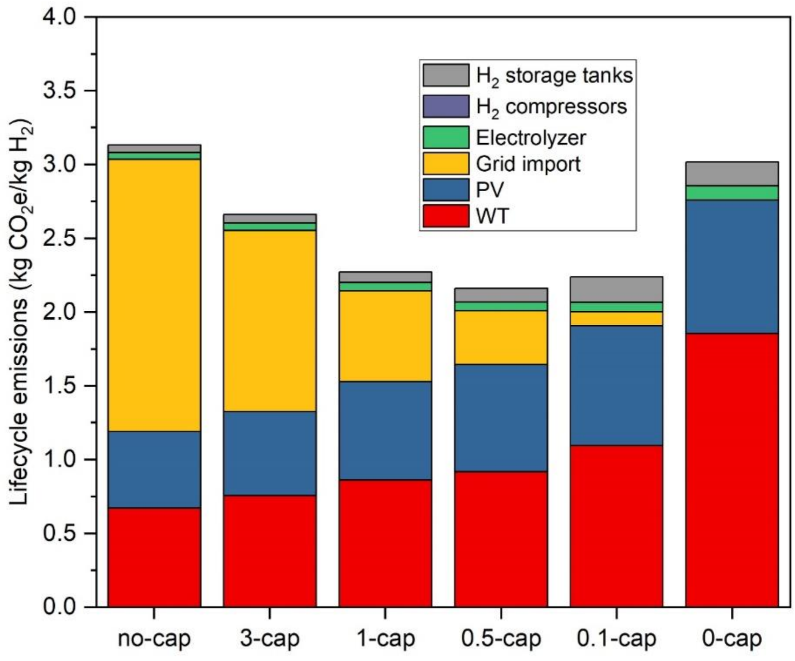

Supplementary Figure 7 | Average lifecycle emissions (including scope 1, scope 2, partial scope 3 upstream emissions and technology manufacturing) of electrolytic hydrogen in Europe in kilograms carbon dioxide equivalent per kilogram of hydrogen (kg CO_2e/kg H_2). When including emissions from producing photovoltaics (PV), wind turbines (WT), electrolyzers, compressors, and hydrogen storage tanks, there is an increase of 1.3 kg CO_2e/kg H_2 in the carbon content of hydrogen under the no-cap scenario, culminating in a lifecycle carbon content of 3.1 kg CO_2e/kg H_2. Conversely, under the 0-cap scenario, these emissions could rise by an average of 3.0 kg CO_2e/kg H_2. Including emissions from the manufacturing of renewable installations increase on average 1.19 kg CO_2e/kg H_2 under the no-cap scenario (56% from WT and 44% from PV) and rise to 2.76 kg CO_2e/kg H_2 under the 0-cap scenario when larger over-capacities are required (67% for WT and 33% for PV). Notwithstanding this addition, there would still be an approximate 75% reduction in emissions compared to steam methane reforming (SMR) technology, even without factoring in the manufacturing emissions for SMR.

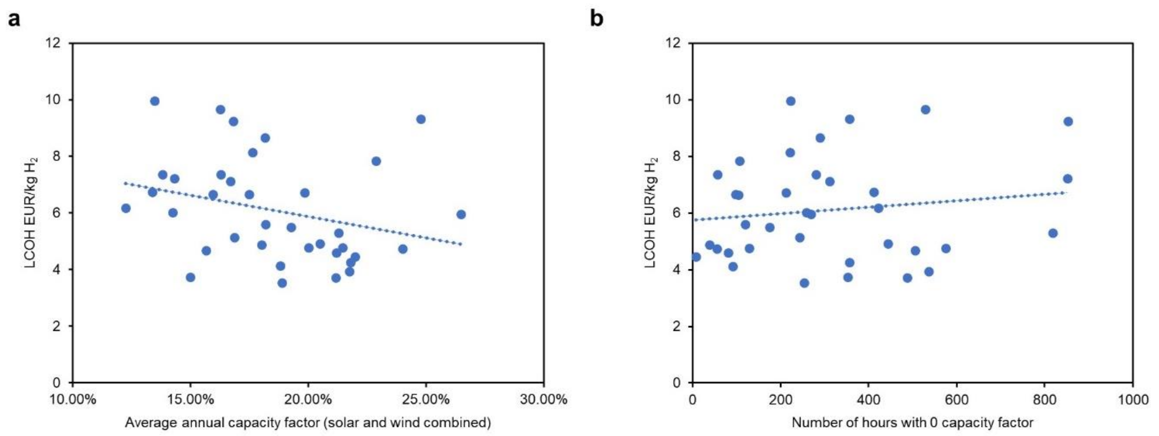

Supplementary Figure 8 | Correlation between weather conditions and the levelized cost of hydrogen (LCOH) in euro per kilogram of hydrogen (EUR/kg H_2). Results under 0-cap and average scenario. **a** weak negative correlation within average annual capacity factor (both solar and wind combined) and LCOH. **b** weak positive correlation within the number of hours with 0 capacity factor and LCOH.

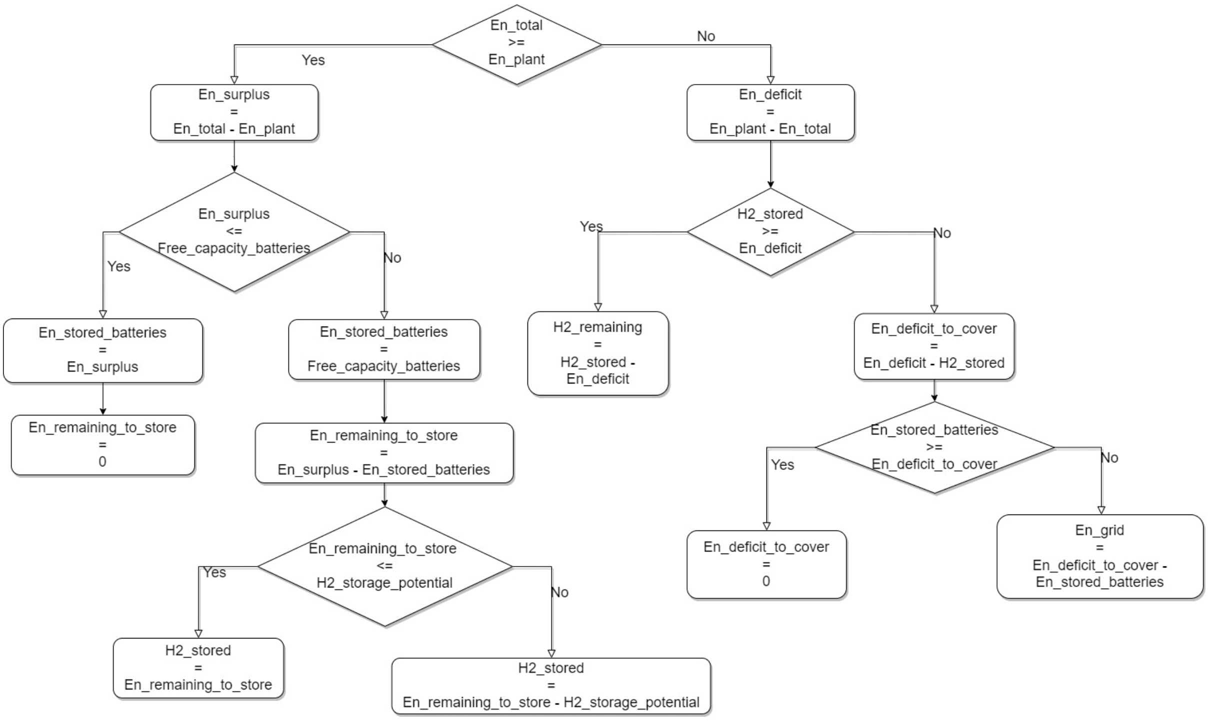

Supplementary Figure 9 | Heuristic optimization model description. En_total represents the combined energy output from solar photovoltaic (PV) panels and wind turbines. En_plant indicates the hourly energy demand necessary to produce 7.5 tons of hydrogen. Free_capacity_batteries denotes the unused and available capacity of the batteries for energy storage at any given time. H2_storage_potential questions whether there is sufficient capacity within the electrolyzers, compressors, and storage tanks to convert excess energy into hydrogen, followed by compression and storage. Lastly, En_grid refers to the energy that is supplied from the electrical grid to the system.

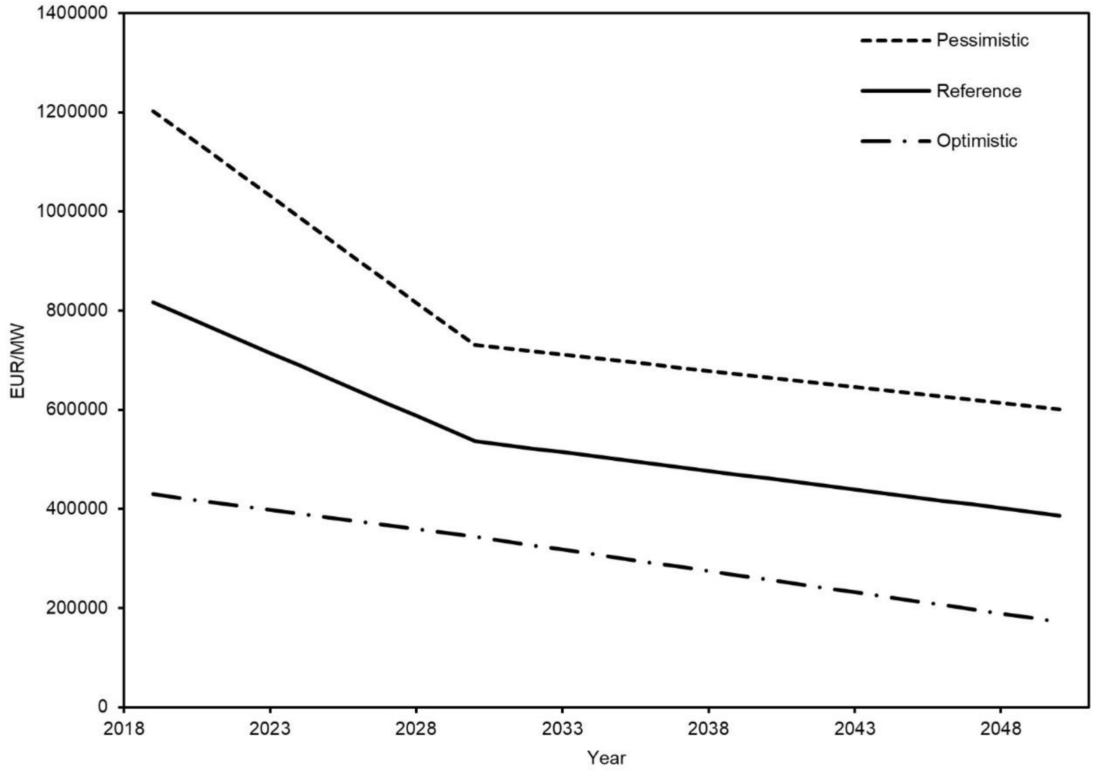

Supplementary Figure 10 | Electrolyzer system cost development (in euro per megawatt or EUR/MW) in pessimistic, reference, and optimistic cases. Authors' elaboration based on data collected from[27]. The International Energy Agency (IEA) provides the techno-economic characteristics of different electrolyzer technologies for 2019 and projections for 2030 and 2050.

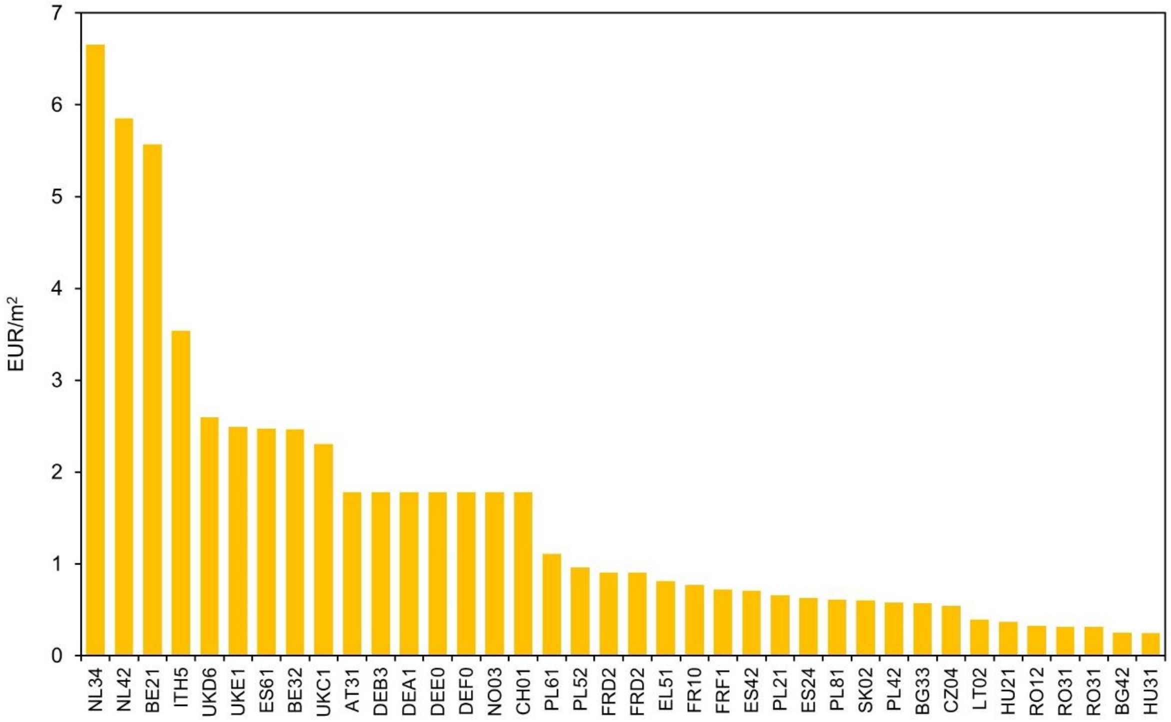

Supplementary Figure 11 | Cost of land in Europe measured in euro per square meters (EUR/m^2). For regions without data, the mean value of the dataset is used. Data collected at NUTS-2 (nomenclature of territorial units for statistics) level from[28]. Regions coded according to the NUTS-2 level of the Nomenclature of Territorial Units for Statistics (NUTS) system.

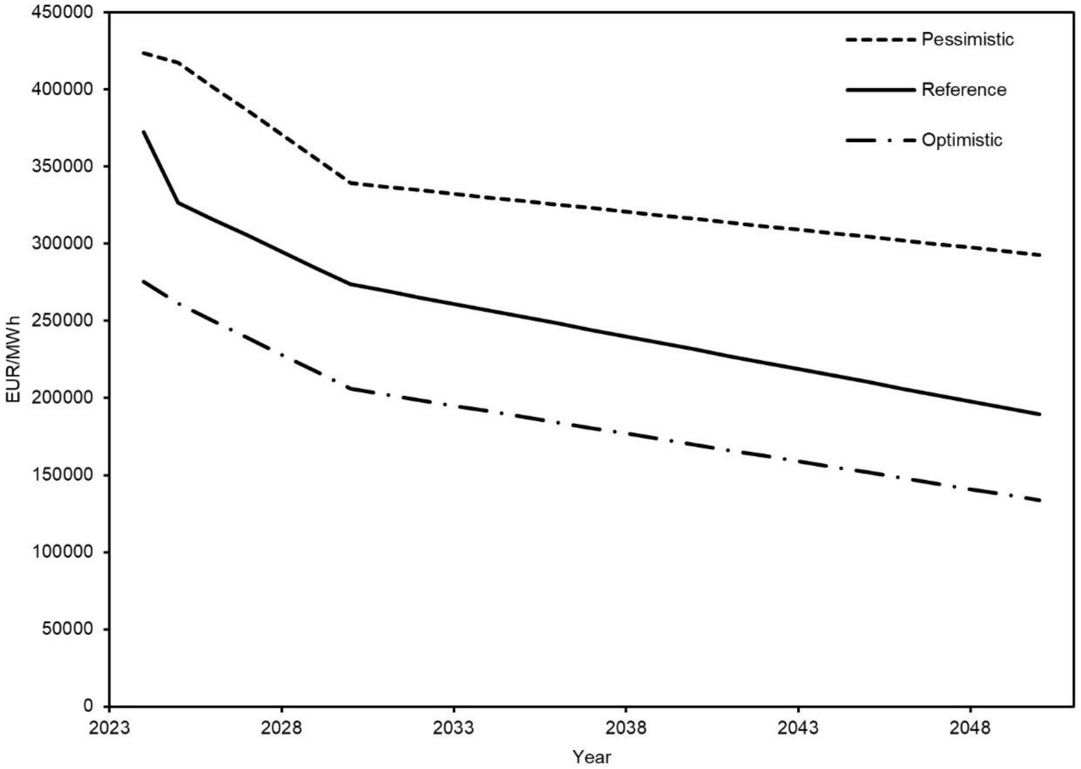

Supplementary Figure 12 | Utility-scale Li-ion batteries system cost projections (in euro per megawatt hour or EUR/MWh) in pessimistic, reference, and optimistic cases. Authors' elaboration based on data collected from National Renewable Energy Laboratory (NREL)[29]. System cost projections as a total system overnight capital cost expressed in units of EUR/MWh. Optimistic, reference, and pessimistic projections are based on the low, median, and higher values collected by NREL from cost projections published in 2019 or later. According to NREL, by 2030, system costs will decrease by 58%, 42%, and 28% in the low, mid, and high cases respectively, and by 2050 decrease by 75%, 57%, and 28%, respectively, compared to 2020.

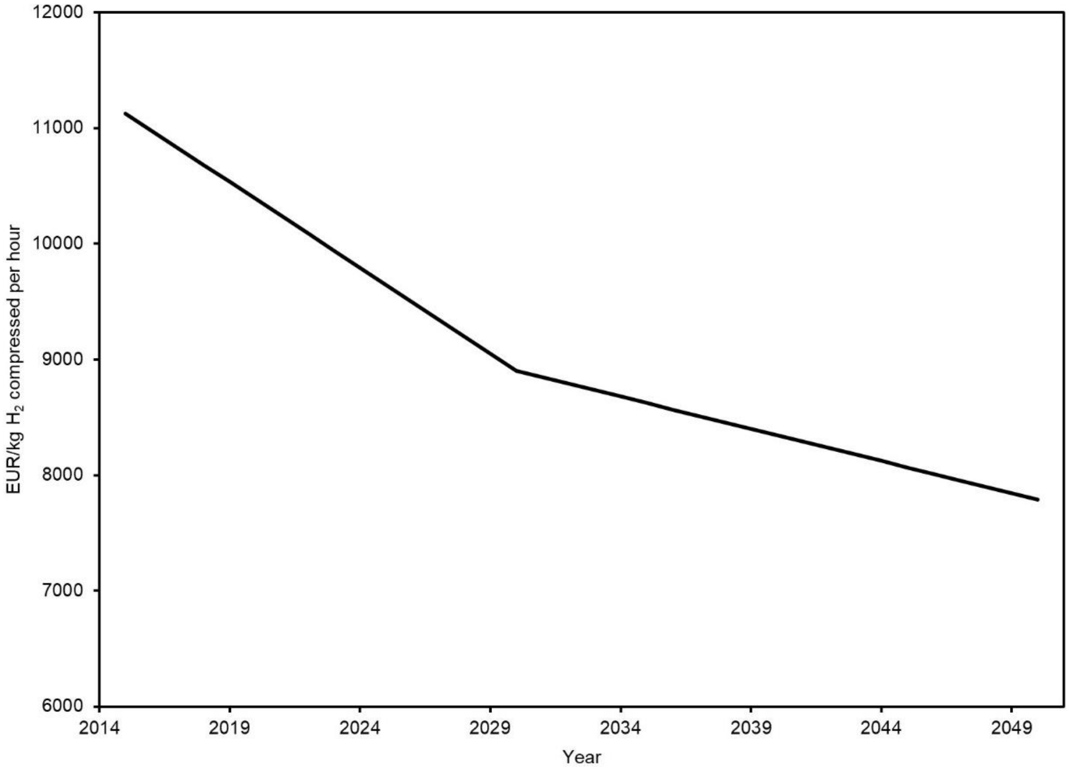

Supplementary Figure 13 | Cost projection hydrogen compressor (up to 900 bar) in euro per kilogram of hydrogen compressed per hour (EUR/kg H_2/h). Authors' elaboration based on data collected from[30,31]. Costs associated with utility-scale dedicated hydrogen compressors are expected to decrease by approximately 30% from the year 2015 to 2050.

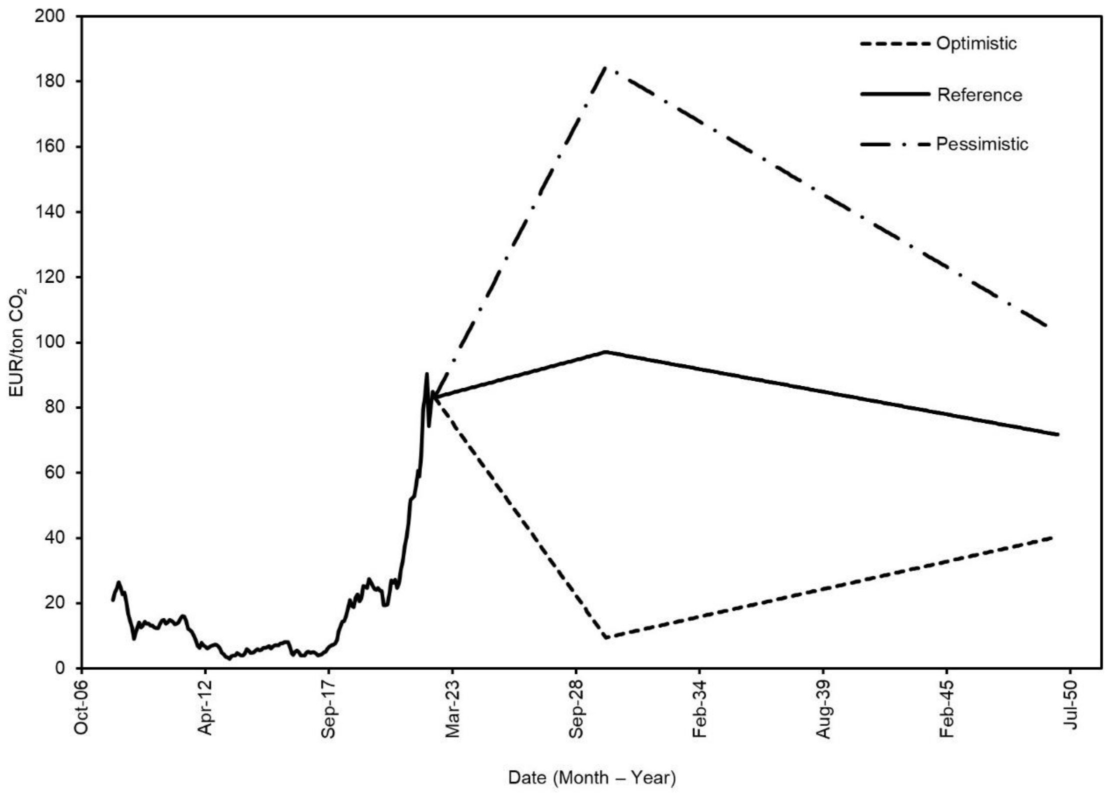

Supplementary Figure 14 | European Union Emission Trading Scheme (EU-ETS) price projection in pessimistic, reference, and optimistic cases. Price ranges from 9 to 185 euro per ton of carbon dioxide (EUR/ton CO_2) in 2030, up from just 2.50 EUR on average in 2020, before narrowing to 40-100 EUR/ton CO_2 in 2050[32].

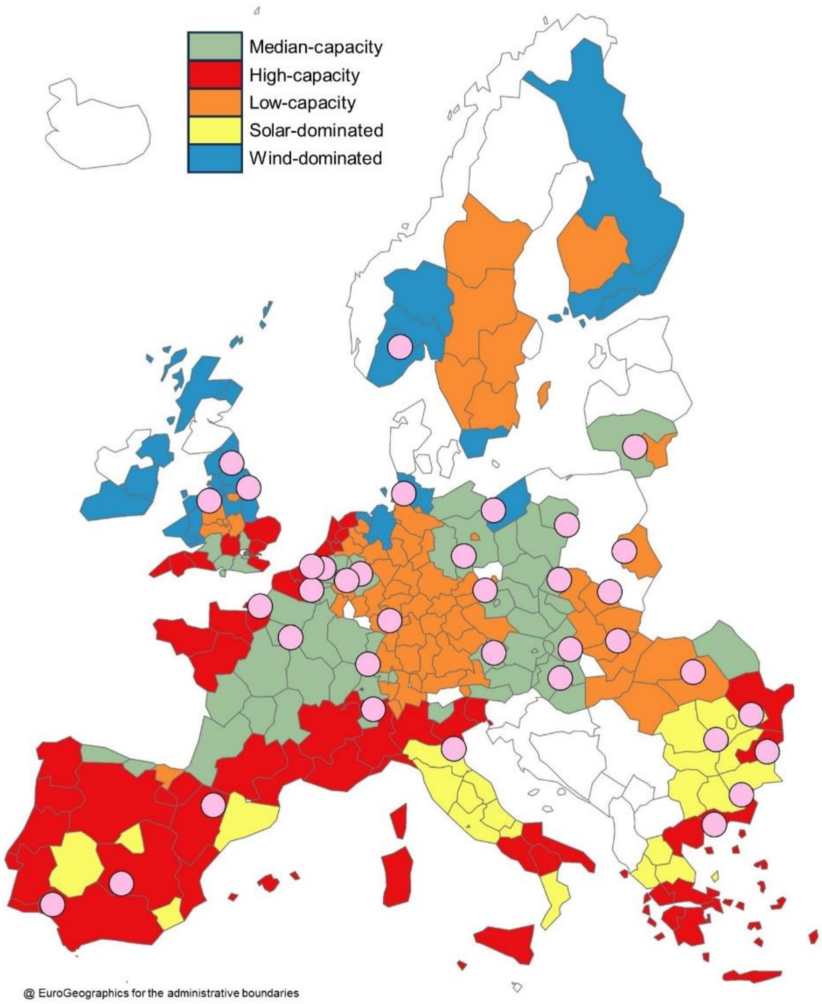

Supplementary Figure 15 | European regions clustered based on their solar and wind capacity factor. Pink dots indicate the location of existing European ammonia plants, pinpointed using data from Yara, Fertiberia, BASF, and Borealis websites, verified against European Commission[33] and Fertilizer Europe[5] databases, with additional steam methane reforming (SMR) hydrogen production sites cross-checked from FCHO[6] (see Supplementary Note 2). Geospatial data for the NUTS-0 (Nomenclature of Territorial Units for Statistics) and NUTS-2 European regions were obtained in the form of shapefiles from Eurostat, the statistical office of the European Union[34]

Country name	Region name	NUTS-2 code	Total land area (km^2)	% of land available (at country level)	Current electricity Consumption (TWh) (national level)	Current electricity Consumption (TWh) (NUTS-2 level)	Renewable potential (TWh) (NUTS-2 level)	Mean capacity factor solar	Mean capacity factor wind	Note
Austria	Upper Austria	AT31	11982	35%	64	18	18.9	12%	21%	
Belgium	Antwerp	BE21	2876	53%	87	15	3.5	12%	24%	
Belgium	Hainaut	BE32	3813	53%	87	10	5.4	12%	23%	
Bulgaria	Severoiztochen	BG33	14487	42%	39	5	49.3	15%	23%	
Bulgaria	Yugoiztochen	BG42	22365	42%	39	8	23.3	16%	14%	
Switzerland	Lake Geneva region	CH01	8719	42%	60	12		13%	30%	
Czech Republic	Severozápad	CZ04	8649	43%	66	7	8.9	11%	15%	
Germany	Düsseldorf	DEA1	5290	45%	493	50	9.8	12%	22%	
Germany	Rheinhessen-Pfalz	DEB3	6852	45%	493	12	8.3	12%	15%	
Germany	Sachsen-Anhalt	DEE0	20452	45%	493	13	48.0	12%	20%	
Germany	Schleswig-Holstein	DEF0	15763	45%	493	16	30.3	12%	34%	
Greece	Eastern Macedonia and Thrace	EL51	14158	46%	51	3	37.2	16%	27%	
Spain	Aragon	ES24	47720	43%	253	7	85.2	18%	24%	
Spain	Castilla-La Mancha	ES42	79463	43%	253	11	85.2	19%	19%	
Spain	Andalusia	ES61	87268	43%	253	43	85.2	20%	22%	
France	Île de France	FR10	12012	41%	476	86	25.6	13%	22%	
France	Upper Normandy	FRD2	12317	41%	476	13	29.6	12%	32%	2 ammonia plants in this region
France	Alsace	FRF1	8280	41%	476	14	10.2	13%	19%	
Croatia	Panonska Hrvatska	HR02	N/A	N/A	N/A	N/A	N/A	N/A	N/A	Capacity factors not available. Plant not included in the analysis
Hungary	Közép-Dunántúl	HU21	11237	52%	43	5	14.2	15%	21%	
Hungary	Észak-Magyarország	HU31	13428	52%	43	6	36.5	15%	12%	
Italy	Emilia-Romagna	ITH5	22446	45%	291	21	110.9	16%	13%	
Lithuania	Vidurio ir vakarų Lietuvos regionas	LT02	55539	41%	12	8	4.5	11%	29%	
Netherlands	Zeeland	NL34	2934	44%	114	2	2.9	12%	38%	

Netherlands	Limburg	NL42	2148	44%	114	7	2.9	12%	20%	
Norway	Sør-Østlandet	NO03	35610	3%	128	22		10%	43%	
Poland	Małopolskie	PL21	15108	46%	168	15	16.2	12%	17%	
Poland	Zachodniopomorskie	PL42	22892	46%	168	7	42.8	12%	29%	
Poland	Opolskie	PL52	9413	46%	168	13	19.2	13%	26%	
Poland	Kujawsko-Pomorskie	PL61	17969	46%	168	9	43.8	12%	28%	
Poland	Lubelskie	PL81	25155	46%	168	9	62.2	12%	24%	
Romania	Centru	RO12	34082	45%	60	6	35.7	14%	10%	
Romania	Macroregiunea trei	RO31	34489	45%	60	12	106.8	14%	17%	2 ammonia plants in this region
Slovakia	Západné Slovensko	SK02	14992	40%	30	9	41.4	14%	19%	
United Kingdom	Tees Valley and Durham	UKC1	3516	60%	306	11	4.9	10%	33%	
United Kingdom	Cheshire	UKD6	2343	60%	306	4	4.0	11%	32%	
United Kingdom	East Riding and North Lincolnshire	UKE1	9438	60%	306	4	12.4	11%	37%	

Supplementary Table 1 | Major European ammonia plants' location and national/regional characteristics. The percentage of land available is derived from the Food and Agriculture Organization of the United Nations (FAOSTAT)[35] using the methodology described by Gabrielli et al.[36]; Current electricity consumption and renewable potential from Kakoulaki et al.,[37]; Capacity factors from European Meteorological derived HIgh resolution RES generation time series (EMHIRES) datasets[38–40] at NUTS-2 (Nomenclature of Territorial Units for Statistics) level. Land area measured in square kilometers (km^2) and electricity demand in terawatt hours (TWh).

Name	Market	System boundary	Emission cap (kg CO_2e/kg H_2)	Source
UK Low Carbon Hydrogen Standard / UK Low Carbon Hydrogen Certificate Scheme	United Kingdom	Well-to-gate	2.4	[41]
Renewable Transportation Fuel Obligation	United Kingdom	Well-to-point of delivery	4	[42]
EU Taxonomy	European Union	Well-to-gate		[2]
Renewable Energy Directive II	European Union	Well-to-wheel	3.4	[1]
Low-carbon fuel standard	California (United States)	Well-to-wheel	1.3-14.1	[43]
Clean Hydrogen Production Tax Credit	United States	Well-to-gate	0.45 – 4	[44]
Clean Hydrogen Investment Tax Credit	Canada	Well-to-gate	0.75 – 4	[45]
France Ordinance No. 2021-167	France	Well-to-gate including manufacturing of technologies	3.38	[46]
Clean Hydrogen and Renewable Hydrogen (China Hydrogen Alliance)	China	Well-to-gate	4.9 – 14.5	[47]
CertifHy	European Union	Well-to-gate	4.4	[48]
Green Hydrogen Standard (Green Hydrogen Organisation)	International	Well-to-gate	0.3 – 1	[49]

Supplementary Table 2 | Existing and planned regulatory frameworks and certifications for hydrogen, ammonia, and hydrogen-based fuels. Adapted from[23]. Emission caps measured in terms of kilogram of carbon dioxide equivalents per kilogram of hydrogen (kg CO_2e/kg H_2).

Emission caps	no-cap			3-cap			1-cap			0.5-cap			0.1-cap			0-cap		
Cases	Optimistic	Reference	Pessimistic	Optimistic	Reference	Pessimistic	Optimistic	Reference	Pessimistic	Optimistic	Reference	Pessimistic	Optimistic	Reference	Pessimistic	Optimistic	Reference	Pessimistic
AT31	2.74	4.07	6.25	2.74	4.07	6.25	2.75	4.08	6.26	2.90	4.17	6.42	3.16	4.47	6.92	3.60	4.86	7.27
BE21	2.30	4.43	7.66	2.30	4.43	7.66	2.76	4.56	7.66	3.19	4.73	7.66	3.82	5.16	7.66	4.89	6.36	8.95
BE32	2.23	4.27	7.34	2.23	4.27	7.34	2.56	4.32	7.34	2.92	4.44	7.34	3.51	4.82	7.34	4.63	6.07	8.66
BG33	2.57	3.95	6.39	2.57	3.95	6.39	2.59	3.95	6.39	2.64	3.95	6.39	2.93	4.15	6.46	3.32	4.60	7.07
BG42	2.82	4.36	6.95	2.82	4.36	6.95	2.84	4.36	6.95	2.91	4.36	6.95	3.11	4.40	6.95	3.43	4.78	7.45
CH01	2.33	3.80	5.93	2.33	3.80	5.93	2.33	3.80	5.93	2.33	3.80	5.93	2.51	3.80	5.93	2.98	4.09	6.12
CZ04	1.80	3.40	6.23	2.50	3.99	6.74	3.19	4.69	7.55	3.47	4.95	7.83	4.08	5.47	8.21	5.00	6.64	9.70
DEA1	3.46	4.76	7.00	3.46	4.76	7.00	3.46	4.76	7.00	3.46	4.76	7.00	3.66	4.83	7.00	4.22	5.54	7.89
DEB3	3.65	5.03	7.57	3.65	5.03	7.57	3.65	5.03	7.57	3.65	5.03	7.57	4.04	5.37	7.86	5.52	7.34	10.39
DEE0	3.74	5.16	7.75	3.74	5.16	7.75	3.74	5.16	7.75	3.74	5.16	7.75	3.99	5.31	7.78	4.57	6.06	8.78
DEF0	2.63	3.63	5.42	2.63	3.63	5.42	2.63	3.63	5.42	2.63	3.63	5.42	2.65	3.64	5.47	6.21	8.29	11.71
EL51	1.98	3.48	5.63	1.98	3.48	5.63	2.27	3.50	5.63	2.42	3.56	5.63	2.68	3.76	5.63	2.85	3.81	5.67
ES24	1.63	3.12	5.62	1.63	3.12	5.62	1.85	3.18	5.62	2.14	3.33	5.62	2.49	3.59	5.69	3.47	5.09	8.50
ES42	1.62	3.26	5.89	1.62	3.26	5.89	1.97	3.37	5.89	2.23	3.49	5.89	2.52	3.69	5.91	2.79	3.88	5.97
ES61	1.62	3.21	5.52	1.62	3.21	5.52	1.95	3.28	5.52	2.20	3.38	5.52	2.53	3.60	5.54	3.78	5.12	7.34
FR10	2.88	4.61	7.49	2.88	4.61	7.49	2.88	4.61	7.49	2.88	4.61	7.49	3.09	4.61	7.49	4.94	7.16	10.84
FRD2	2.50	3.91	6.21	2.50	3.91	6.21	2.50	3.91	6.21	2.50	3.91	6.21	2.63	3.91	6.21	3.17	4.32	6.61
FRF1	2.95	4.75	7.68	2.95	4.75	7.68	2.95	4.75	7.68	2.96	4.75	7.68	3.17	4.75	7.68	6.82	10.24	15.66
HU21	1.67	3.12	5.99	1.67	3.12	5.99	2.13	3.49	6.17	2.49	3.78	6.33	2.95	4.22	6.76	3.53	4.89	7.64
HU31	1.67	3.21	6.53	1.67	3.21	6.53	2.49	4.12	7.37	2.96	4.59	7.85	4.21	5.78	8.96	8.66	11.70	16.86
ITH5	3.74	5.35	8.23	3.74	5.35	8.23	3.74	5.35	8.23	3.74	5.35	8.23	4.01	5.68	8.97	6.14	8.13	11.83
LT02	2.17	3.58	5.75	2.17	3.58	5.75	2.17	3.58	5.75	2.17	3.58	5.75	2.53	3.68	5.77	5.37	7.10	10.29
NL34	2.17	3.36	5.14	2.18	3.38	5.19	2.70	3.86	5.84	2.92	4.07	6.09	3.31	4.42	6.44	6.37	8.97	14.56
NL42	2.36	3.88	6.10	2.62	4.16	6.70	3.60	5.14	8.03	3.94	5.50	8.54	4.61	6.07	8.98	5.90	7.70	11.04
NO03	0.72	1.99	4.71	0.72	1.99	4.71	0.72	1.99	4.71	0.72	1.99	4.71	0.90	1.99	4.71	4.64	6.68	9.76
PL21	1.83	3.40	6.20	2.65	4.11	6.79	3.15	4.54	7.17	3.29	4.66	7.34	4.49	6.31	9.58	6.27	8.64	12.61
PL42	1.69	2.98	5.26	2.01	3.18	5.30	2.39	3.48	5.48	2.54	3.63	5.61	2.83	3.82	5.73	2.90	3.89	5.78

PL52	1.77	3.10	5.49	2.14	3.36	5.55	2.55	3.71	5.86	2.72	3.89	6.02	3.02	4.12	6.18	4.70	6.83	9.97
PL61	1.73	3.05	5.32	2.08	3.26	5.35	2.45	3.53	5.52	2.60	3.67	5.67	2.97	4.12	6.18	3.39	4.64	6.87
PL81	1.78	3.14	5.61	2.22	3.51	5.84	2.76	4.00	6.35	2.94	4.21	6.60	3.37	4.60	6.93	3.51	4.70	7.00
RO12	2.83	4.88	8.19	2.86	4.88	8.19	3.14	4.89	8.19	3.41	5.04	8.23	4.34	6.24	10.55	6.14	8.74	14.93
RO31	2.70	4.19	6.69	2.70	4.19	6.69	2.81	4.19	6.69	2.87	4.19	6.69	3.07	4.37	6.88	3.55	4.85	7.41
SK02	3.01	4.60	7.47	3.01	4.60	7.47	3.01	4.60	7.47	3.06	4.60	7.47	4.45	6.56	10.93	9.00	12.61	21.97
UKC1	2.85	4.34	6.60	2.85	4.34	6.60	2.85	4.34	6.60	2.86	4.34	6.60	3.05	4.34	6.60	3.18	4.34	6.58
UKD6	2.95	4.84	7.57	2.95	4.84	7.57	2.95	4.84	7.57	2.99	4.84	7.57	3.55	5.10	7.57	3.90	5.35	8.03
UKE1	2.74	4.26	6.59	2.74	4.26	6.59	2.74	4.26	6.59	2.78	4.26	6.59	3.01	4.26	6.59	3.12	4.27	6.57
Min	0.72	1.99	4.71	0.72	1.99	4.71	0.72	1.99	4.71	0.72	1.99	4.71	0.90	1.99	4.71	2.79	3.81	5.67
Mean	2.38	3.90	6.44	2.48	3.97	6.50	2.70	4.13	6.65	2.84	4.23	6.73	3.26	4.58	7.11	4.62	6.34	9.56
Max	3.74	5.35	8.23	3.74	5.35	8.23	3.74	5.35	8.23	3.94	5.50	8.54	4.61	6.56	10.93	9.00	12.61	21.97

Supplementary Table 3 | Levelized cost of hydrogen (LCOH) in euro per kilogram of hydrogen (EUR/kg H_2) across European ammonia plants for different cases (optimistic, reference, and pessimistic) and emission caps.

Emission caps	no-cap			3-cap			1-cap			0.5-cap			0.1-cap			0-cap
Cases	Optimistic	Reference	Pessimistic	Optimistic	Reference	Pessimistic	Optimistic	Reference	Pessimistic	Optimistic	Reference	Pessimistic	Optimistic	Reference	Pessimistic	Optimistic - Reference - Pessimistic
AT31	91%	91%	90%	91%	91%	90%	92%	92%	92%	96%	96%	96%	99%	99%	99%	100%
BE21	78%	86%	100%	78%	86%	100%	92%	92%	100%	96%	96%	100%	99%	99%	100%	100%
BE32	85%	87%	99%	85%	87%	99%	92%	92%	99%	96%	96%	99%	99%	99%	99%	100%
BG33	86%	97%	98%	86%	97%	98%	92%	97%	98%	96%	97%	98%	99%	99%	99%	100%
BG42	80%	97%	99%	80%	97%	99%	92%	97%	99%	96%	97%	99%	99%	99%	99%	100%
CH01	97%	100%	100%	97%	100%	100%	97%	100%	100%	97%	100%	100%	99%	100%	100%	100%
CZ04	34%	49%	49%	75%	75%	75%	92%	92%	92%	96%	96%	96%	99%	99%	99%	100%
DEA1	97%	98%	99%	97%	98%	99%	97%	98%	99%	97%	98%	99%	99%	99%	99%	100%
DEB3	97%	97%	97%	97%	97%	97%	97%	97%	97%	97%	97%	97%	99%	99%	99%	100%
DEE0	97%	98%	99%	97%	98%	99%	97%	98%	99%	97%	98%	99%	99%	99%	99%	100%
DEF0	99%	99%	99%	99%	99%	99%	99%	99%	99%	99%	99%	99%	99%	99%	99%	100%
EL51	77%	87%	99%	77%	87%	99%	92%	92%	99%	96%	96%	99%	99%	99%	99%	100%
ES24	83%	87%	96%	83%	87%	96%	92%	92%	96%	96%	96%	96%	99%	99%	99%	100%
ES42	83%	86%	98%	83%	86%	98%	92%	92%	98%	96%	96%	98%	99%	99%	99%	100%
ES61	83%	88%	98%	83%	88%	98%	92%	92%	98%	96%	96%	98%	99%	99%	99%	100%
FR10	96%	99%	100%	96%	99%	100%	96%	99%	100%	96%	99%	100%	99%	99%	100%	100%
FRD2	98%	99%	100%	98%	99%	100%	98%	99%	100%	98%	99%	100%	99%	99%	100%	100%
FRF1	95%	99%	100%	95%	99%	100%	95%	99%	100%	96%	99%	100%	99%	99%	100%	100%
HU21	82%	84%	85%	82%	84%	85%	92%	92%	92%	96%	96%	96%	99%	99%	99%	100%
HU31	78%	80%	81%	78%	80%	81%	92%	92%	92%	96%	96%	96%	99%	99%	99%	100%
ITH5	95%	98%	98%	95%	98%	98%	95%	98%	98%	96%	98%	98%	99%	99%	99%	100%
LT02	96%	98%	99%	96%	98%	99%	96%	98%	99%	96%	98%	99%	99%	99%	99%	100%
NL34	74%	72%	71%	75%	75%	75%	92%	92%	92%	96%	96%	96%	99%	99%	99%	100%
NL42	58%	61%	58%	75%	75%	75%	92%	92%	92%	96%	96%	96%	99%	99%	99%	100%

NO03	99%	99%	100%	99%	99%	100%	99%	99%	100%	99%	99%	100%	99%	99%	100%	100%
PL21	17%	36%	37%	75%	75%	75%	92%	92%	92%	96%	96%	96%	99%	99%	99%	100%
PL42	45%	52%	60%	75%	75%	75%	92%	92%	92%	96%	96%	96%	99%	99%	99%	100%
PL52	40%	49%	59%	75%	75%	75%	92%	92%	92%	96%	96%	96%	99%	99%	99%	100%
PL61	43%	50%	65%	75%	75%	75%	92%	92%	92%	96%	96%	96%	99%	99%	99%	100%
PL81	38%	47%	51%	75%	75%	75%	92%	92%	92%	96%	96%	96%	99%	99%	99%	100%
RO12	72%	89%	92%	75%	89%	92%	92%	92%	92%	96%	96%	96%	99%	99%	99%	100%
RO31	79%	96%	98%	79%	96%	98%	92%	96%	98%	96%	96%	98%	99%	99%	99%	100%
SK02	93%	97%	96%	93%	97%	96%	93%	97%	96%	96%	97%	96%	99%	99%	99%	100%
UKC1	95%	100%	100%	95%	100%	100%	95%	100%	100%	96%	100%	100%	99%	100%	100%	100%
UKD6	93%	96%	100%	93%	96%	100%	93%	96%	100%	96%	96%	100%	99%	99%	100%	100%
UKE1	93%	100%	100%	93%	100%	100%	93%	100%	100%	96%	100%	100%	99%	100%	100%	100%

Min	17%	36%	37%	75%	75%	75%	92%	92%	92%	96%	96%	96%	99%	99%	99%	100%
Mean	79%	85%	88%	86%	90%	92%	93%	95%	96%	96%	97%	98%	99%	99%	99%	100%
Max	99%	100%	100%	99%	100%	100%	99%	100%	100%	99%	100%	100%	99%	100%	100%	100%

Supplementary Table 4 | Emission reduction across European ammonia plants for different scenarios, cases (optimistic, reference, and pessimistic) and emission caps.

Emission caps	no-cap			3-cap			1-cap			0.5-cap			0.1-cap			0-cap		
Cases	Optimistic	Reference	Pessimistic	Optimistic	Reference	Pessimistic	Optimistic	Reference	Pessimistic	Optimistic	Reference	Pessimistic	Optimistic	Reference	Pessimistic	Optimistic	Reference	Pessimistic
AT31	68.13	189.40	392.44	68.13	189.40	392.44	68.30	188.73	387.66	77.92	188.73	384.04	97.39	207.66	413.23	133.29	238.48	438.80
BE21	32.07	235.57	474.03	32.07	235.57	474.03	69.40	232.44	474.03	103.71	237.67	474.03	153.06	265.51	474.03	240.93	363.74	579.17
BE32	22.56	217.46	447.90	22.56	217.46	447.90	50.73	210.49	447.90	79.68	211.95	447.90	127.08	237.27	447.90	219.01	339.04	554.64
BG33	55.71	166.64	373.98	55.71	166.64	373.98	53.47	166.64	373.98	55.62	166.64	373.98	77.77	180.88	374.85	110.12	216.93	422.27
BG42	85.70	203.28	416.14	85.70	203.28	416.14	76.77	203.28	416.14	79.27	203.28	416.14	92.96	201.56	416.14	118.81	231.58	454.00
CH01	28.73	150.67	327.97	28.73	150.67	327.97	28.73	150.67	327.97	28.73	150.67	327.97	42.58	150.67	327.97	81.78	173.91	343.45
CZ04	0.00	240.72	718.64	55.32	221.13	527.05	108.02	244.20	504.72	128.19	256.78	506.94	174.69	291.30	521.66	250.29	386.91	641.57
DEA1	126.13	234.12	420.51	126.13	234.12	420.51	126.13	234.12	420.51	126.13	234.12	420.51	139.68	238.22	420.30	185.30	295.09	491.02
DEB3	142.32	261.32	479.50	142.32	261.32	479.50	142.32	261.32	479.50	142.32	261.32	479.50	171.62	283.03	492.61	293.47	444.63	698.94
DEE0	150.00	270.22	486.43	150.00	270.22	486.43	150.00	270.22	486.43	150.00	270.22	486.43	166.93	278.02	485.44	214.00	338.66	564.82
DEF0	53.46	137.60	288.99	53.46	137.60	288.99	53.46	137.60	288.99	53.46	137.60	288.99	54.79	137.54	291.26	350.82	523.97	809.06
EL51	0.00	141.14	306.07	0.00	141.14	306.07	24.36	136.53	306.07	36.96	135.37	306.07	56.92	147.64	305.44	70.50	150.54	305.58
ES24	0.00	106.68	312.87	0.00	106.68	312.87	0.00	107.25	312.87	12.27	115.38	312.87	41.37	133.54	309.89	122.61	257.52	541.97
ES42	0.00	122.18	331.64	0.00	122.18	331.64	0.00	124.25	331.64	19.92	129.77	331.64	43.29	142.30	328.44	66.10	156.71	330.88
ES61	0.00	115.64	298.81	0.00	115.64	298.81	0.00	116.07	298.81	17.54	119.95	298.81	44.33	134.10	297.46	148.11	259.63	444.75
FR10	76.69	218.60	458.12	76.69	218.60	458.12	76.69	218.60	458.12	76.53	218.60	458.12	91.79	218.60	458.12	245.09	429.66	736.83
FRD2	42.40	160.10	351.27	42.40	160.10	351.27	42.40	160.10	351.27	42.40	160.10	351.27	52.56	160.10	351.27	97.22	193.65	383.78
FRF1	83.58	230.87	474.27	83.58	230.87	474.27	83.58	230.87	474.27	83.87	230.87	474.27	98.66	230.74	474.27	401.50	686.53	1138.68
HU21	0.00	110.53	392.01	0.00	110.53	392.01	11.69	135.56	378.95	42.45	154.73	376.27	79.58	186.47	399.94	127.87	241.20	470.05
HU31	0.00	126.12	466.07	0.00	126.12	466.07	44.45	192.51	488.48	83.81	225.15	509.08	185.85	317.90	584.59	554.67	808.24	1238.47
ITH5	152.56	285.78	528.00	152.56	285.78	528.00	152.56	285.78	528.00	151.41	285.78	528.00	169.17	309.50	585.81	344.64	510.81	819.06
LT02	14.66	135.22	315.73	14.66	135.22	315.73	14.66	135.22	315.73	14.66	135.22	315.73	44.50	141.49	316.67	280.67	424.62	691.02
NL34	19.41	158.13	368.59	19.78	153.19	354.55	64.09	169.37	348.80	80.32	180.04	355.54	109.86	203.45	373.43	364.51	581.04	1046.94

NL42	52.07	256.28	585.28	69.29	239.50	522.38	145.76	285.83	548.38	168.93	304.56	568.54	219.14	342.35	586.93	325.06	474.99	753.24
NO03	0.00	0.00	226.40	0.00	0.00	226.40	0.00	0.00	226.40	0.00	0.00	226.40	0.00	0.00	226.40	220.38	389.97	646.33
PL21	0.00	325.56	946.27	72.06	234.08	531.97	104.10	230.76	469.72	111.98	231.35	464.37	208.87	362.33	636.95	355.97	553.35	884.04
PL42	0.00	156.26	451.31	1.49	131.65	366.85	35.64	134.10	316.52	46.92	141.34	314.29	69.39	153.14	313.44	74.61	157.27	314.84
PL52	0.00	186.62	491.14	15.46	151.03	394.48	49.55	155.50	350.50	62.44	164.73	349.78	85.99	177.92	351.20	224.75	402.35	663.94
PL61	0.00	176.74	422.95	8.73	139.57	372.17	41.12	139.05	320.41	52.12	145.27	318.87	81.20	178.36	350.84	115.85	220.19	405.98
PL81	0.00	201.95	590.28	24.85	167.28	426.42	68.80	181.84	395.20	82.17	192.47	400.08	115.43	218.56	414.65	125.80	225.13	416.74
RO12	95.27	270.12	558.28	95.61	270.12	558.28	104.06	263.06	558.28	122.44	264.64	541.56	196.96	356.31	718.43	345.15	561.42	1077.56
RO31	74.40	190.04	400.02	74.40	190.04	400.02	73.25	190.04	400.02	76.00	190.04	400.02	90.15	199.22	410.37	129.06	237.42	451.09
SK02	91.02	223.80	472.30	91.02	223.80	472.30	91.02	223.80	472.30	92.19	223.80	472.30	205.93	383.04	750.34	583.68	884.53	1664.06
UKC1	74.80	195.49	383.28	74.80	195.49	383.28	74.80	195.49	383.28	74.54	195.49	383.28	88.49	195.49	383.28	98.18	195.19	381.28
UKD6	85.26	246.13	466.46	85.26	246.13	466.46	85.26	246.13	466.46	86.32	246.13	466.46	129.91	260.21	466.46	158.16	279.01	502.78
UKE1	66.33	189.33	382.61	66.33	189.33	382.61	66.33	189.33	382.61	67.56	189.33	382.61	85.19	189.33	382.61	93.53	189.54	380.61
Min	0.00	0.00	226.40	0.00	0.00	226.40	0.00	0.00	226.40	0.00	0.00	226.40	0.00	0.00	226.40	66.10	150.54	305.58
Mean	47.04	189.90	439.07	52.48	182.54	409.11	66.15	187.41	402.53	75.85	191.64	403.13	108.14	217.05	428.96	218.65	361.76	630.23
Max	152.56	325.56	946.27	152.56	285.78	558.28	152.56	285.83	558.28	168.93	304.56	568.54	219.14	383.04	750.34	583.68	884.53	1664.06

Supplementary Table 5 | Abatement cost (AC) in euro per ton of carbon dioxide equivalent (EUR/t CO_2e) abated across European ammonia plants for different emission caps and cases (optimistic, reference, and pessimistic). If the AC is negative, indicating that the levelized cost of hydrogen (LCOH) for electrolytic hydrogen is lower than that of steam methane reforming (SMR), the AC value is adjusted to zero.

Input parameter	Description	Unit	Value			Note
			Pessimistic	Reference	Optimistic	
c_{PV}	Total system cost utility-scale PV	EUR/MW	1,612,776	649,881	414,862	
c_{WT}	Total system cost utility-scale WT	EUR/MW	1,719,039	1,064,606	822,797	
c_{EL}	Total system cost ALK electrolyzer	EUR/MW	1,669,387	1,164,612	659,837	First installation in 2024
			361,836 in 2031	257,151 in 2033	154,613 in 2034	For the electrolyzer, the 2024 installation also includes project costs, while the following installations refer only to the stack component.
			329,625 in 2041	215,813 in 2044	107,370 in 2045	
p_{E}	Price grid electricity for industrial user	EUR/MWh	697	105	9	
γ_{E}	Carbon intensity grid electricity	kg CO_2e/MWh	244	85	3	

Supplementary Table 6 | Input parameters for robustness analysis considering optimistic, reference, and pessimistic cases. Units: euro: EUR; megawatt: MW; megawatt hour: MWh; kilograms: kg; carbon dioxide equivalent: CO_2e.

Parameter tested	Emission caps	Case	High capacity	Low capacity	Median capacity	Solar-dominated	Wind-dominated	Average	High capacity	Low capacity	Median capacity	Solar-dominated	Wind-dominated	Average
			Difference in % compared to the reference case						LCOH variation in EUR					
Price grid electricity	no-cap	Pessimistic	7.8%	39.2%	19.5%	20.5%	17.7%	20.9%	€ 2.35	€ 6.66	€ 4.42	€ 4.16	€ 2.68	€ 4.05
		Optimistic	-66.7%	-81.0%	-77.0%	-75.7%	-66.8%	-73.4%						
	1-cap	Pessimistic	7.8%	27.8%	19.5%	20.5%	17.7%	18.7%	€ 1.25	€ 2.88	€ 2.08	€ 2.02	€ 1.74	€ 1.99
		Optimistic	-31.7%	-20.0%	-25.9%	-26.1%	-37.3%	-28.2%						
	0.1-cap	Pessimistic	4.1%	10.5%	12.9%	7.6%	10.1%	9.0%	€ 0.26	€ 0.85	€ 0.75	€ 0.49	€ 0.46	€ 0.56
		Optimistic	-3.7%	-1.7%	-2.5%	-2.5%	-3.6%	-2.8%						
	0-cap	Pessimistic	0.0%	0.0%	0.0%	0.0%	0.0%	0.0%	€ -	€ -	€ -	€ -	€ -	€ -
		Optimistic	0.0%	0.0%	0.0%	0.0%	0.0%	0.0%						
Alkaline electrolyzer price, efficiency, lifetime, maintenance	no-cap	Pessimistic	20.3%	10.6%	16.1%	25.9%	18.9%	18.4%	€ 1.23	€ 1.20	€ 1.52	€ 2.10	€ 1.17	€ 1.44
		Optimistic	-18.6%	-11.0%	-17.1%	-22.5%	-17.9%	-17.4%						
	1-cap	Pessimistic	20.3%	17.3%	17.2%	25.9%	18.9%	19.9%	€ 1.23	€ 2.05	€ 1.57	€ 2.10	€ 1.17	€ 1.62
		Optimistic	-18.6%	-16.5%	-17.1%	-22.5%	-17.9%	-18.6%						
	0.1-cap	Pessimistic	21.4%	17.4%	20.1%	22.7%	21.5%	20.6%	€ 1.31	€ 2.34	€ 1.84	€ 2.14	€ 1.38	€ 1.80
		Optimistic	-18.6%	-16.1%	-18.0%	-21.6%	-19.1%	-18.7%						
	0-cap	Pessimistic	18.5%	16.4%	15.8%	26.1%	23.9%	20.1%	€ 1.33	€ 2.86	€ 3.57	€ 2.48	€ 4.44	€ 2.94
		Optimistic	-17.7%	-15.6%	-24.9%	-21.7%	-26.6%	-21.3%						
Photovoltaic price	no-cap	Pessimistic	8.0%	5.9%	9.5%	23.4%	0.1%	9.4%	€ 0.36	€ 0.45	€ 0.63	€ 1.32	€ 0.05	€ 0.56
		Optimistic	-3.4%	-2.2%	-4.2%	-7.0%	-1.6%	-3.7%						
	1-cap	Pessimistic	8.0%	17.3%	10.5%	23.7%	0.1%	11.9%	€ 0.36	€ 1.39	€ 0.67	€ 1.33	€ 0.05	€ 0.76
		Optimistic	-3.4%	-5.7%	-4.2%	-7.0%	-1.6%	-4.4%						
	0.1-cap	Pessimistic	11.3%	14.9%	16.0%	19.8%	6.1%	13.6%	€ 0.49	€ 1.29	€ 0.98	€ 1.25	€ 0.32	€ 0.87
		Optimistic	-3.6%	-3.7%	-4.2%	-6.0%	-3.5%	-4.2%						
	0-cap	Pessimistic	6.4%	7.6%	1.2%	26.4%	0.0%	8.3%	€ 0.35	€ 0.85	€ 0.65	€ 1.73	€ -	€ 0.72
		Optimistic	-3.2%	-1.9%	-6.2%	-7.0%	0.0%	-3.7%						
Wind turbine price	no-cap	Pessimistic	10.2%	3.4%	8.6%	0.0%	10.5%	6.5%	€ 0.45	€ 0.28	€ 0.58	€ 0.01	€ 0.48	€ 0.36
		Optimistic	-4.2%	-1.7%	-4.1%	-0.1%	-4.6%	-3.0%						
	1-cap	Pessimistic	10.2%	6.2%	8.7%	0.0%	10.5%	7.1%						

		Optimistic	-4.2%	-2.9%	-4.1%	-0.1%	-4.6%	-3.2%	€ 0.45	€ 0.54	€ 0.59	€ 0.01	€ 0.48	€ 0.41
	0.1-cap	Pessimistic	10.7%	14.5%	12.2%	3.0%	12.4%	10.5%	€ 0.49	€ 1.39	€ 0.82	€ 0.31	€ 0.59	€ 0.72
		Optimistic	-4.3%	-5.4%	-4.6%	-3.4%	-5.2%	-4.6%						
	0-cap	Pessimistic	14.2%	19.2%	11.1%	1.5%	12.7%	11.7%	€ 0.74	€ 2.37	€ 1.67	€ 0.17	€ 1.66	€ 1.32
		Optimistic	-6.0%	-7.3%	-8.0%	-1.9%	-6.2%	-5.9%						
Carbon intensity grid electricity	no-cap	Pessimistic	0.0%	0.0%	0.0%	0.0%	0.0%	0.0%	€ -	€ -	€ -	€ -	€ (0.00)	€ (0.00)
		Optimistic	0.0%	0.0%	0.0%	0.0%	0.0%	0.0%						
	1-cap	Pessimistic	0.0%	6.9%	1.6%	3.6%	1.8%	2.8%	€ 0.00	€ 0.91	€ 0.07	€ 0.16	€ 0.06	€ 0.24
		Optimistic	0.0%	-8.2%	0.0%	0.0%	0.0%	-1.6%						
	0.1-cap	Pessimistic	1.5%	15.4%	9.5%	3.7%	4.6%	6.9%	€ 0.16	€ 2.47	€ 0.72	€ 0.70	€ 0.37	€ 0.89
		Optimistic	-3.4%	-20.0%	-5.5%	-10.7%	-6.5%	-9.2%						
	0-cap	Pessimistic	0.0%	0.0%	0.0%	0.0%	0.0%	0.0%	€ -	€ -	€ -	€ -	€ -	€ -
		Optimistic	0.0%	0.0%	0.0%	0.0%	0.0%	0.0%						

Supplementary Table 7 | Robustness analysis results in optimistic and pessimistic cases. Relative contribution (in %) and absolute values (euro or EUR). LCOH: levelized cost of hydrogen measured in EUR/kilogram of hydrogen (EUR/kg H_2).

Emission cap	Electrolyzer type	High-capacity	Low-capacity	Median-capacity	Solar-dominated	Wind-dominated	MIN	MEAN	MAX
no-cap	SOE	35%	7%	16%	26%	30%	7%	23%	35%
1-cap	SOE	35%	20%	23%	35%	31%	20%	29%	35%
0.1-cap	SOE	43%	27%	36%	38%	42%	27%	37%	43%
0-cap	SOE	35%	24%	12%	41%	15%	12%	25%	41%
no-cap	ML	14%	28%	17%	7%	14%	7%	16%	28%
1-cap	ML	14%	20%	17%	7%	14%	7%	14%	20%
0.1-cap	ML	11%	22%	17%	6%	14%	6%	14%	22%
0-cap	ML	14%	17%	-1%	2%	-1%	-1%	6%	17%

Supplementary Table 8 | Impact of electrolyzer technology selection on the levelized cost of hydrogen (LCOH) compared to reference alkaline electrolyzer (ALK) in percentage. ML: membraneless electrolyzer; SOE: solid oxide electrolyzer.

Emission cap	Case	High-capacity	Low-capacity	Median-capacity	Solar-dominated	Wind-dominated	Average
no-cap	Pessimistic	1.41%	7.17%	1.72%	2.06%	3.01%	3.07%
	Reference	0.87%	4.28%	1.02%	1.23%	1.87%	1.85%
	Optimistic	0.31%	1.34%	0.32%	0.39%	0.63%	0.60%
1-cap	Pessimistic	1.41%	2.41%	1.72%	2.06%	3.01%	2.12%
	Reference	0.87%	1.42%	1.02%	1.23%	1.87%	1.28%
	Optimistic	0.31%	0.44%	0.32%	0.39%	0.63%	0.42%
0.1-cap	Pessimistic	0.44%	0.21%	0.30%	0.30%	0.43%	0.34%
	Reference	0.26%	0.12%	0.18%	0.18%	0.25%	0.20%
	Optimistic	0.08%	0.04%	0.06%	0.06%	0.08%	0.06%
0-cap	Pessimistic	0.00%	0.00%	0.00%	0.00%	0.00%	0.00%
	Reference	0.00%	0.00%	0.00%	0.00%	0.00%	0.00%
	Optimistic	0.00%	0.00%	0.00%	0.00%	0.00%	0.00%

Supplementary Table 9 | Impact of the European Union Emission Trading Scheme (EU-ETS) on the levelized cost of hydrogen (LCOH) in %. EU-ETS price: Pessimistic = 145 euro per ton of carbon dioxide equivalent (EUR/t CO_2e); reference = 86 EUR/t CO_2e; optimistic = 27 EUR/t CO_2e.

Representative region	Emission-cap			
	No-cap	1-cap	0.1-cap	0-cap
High-capacity	-5%	-5%	-9%	-19%
Low-capacity	5%	-4%	-14%	-32%
Median-capacity	-6%	-6%	-11%	-46%
Solar-dominated	-3%	-3%	-13%	-19%
Wind-dominated	-16%	-16%	-22%	-46%
Average	-5%	-7%	-14%	-32%

Supplementary Table 10 | Impact of plant flexibility ($\delta_{\mathrm{H2}} D_{\mathrm{H2},t}$) on the levelized cost of hydrogen (LCOH). LCOH variation in % compared to the reference case (continuous ammonia production).

NUTS-2	Grid electricity demand/NUTS-2 electricity demand (1-cap)			Renewable demand/NUTS-2 renewable theoretical potential (1-cap)			Renewable demand/NUTS-2 renewable theoretical potential (0-cap)			Area required/NUTS-2 area available (1-cap)			Area required/NUTS-2 area available (0-cap)		
	Optimistic	Reference	Pessimistic	Optimistic	Reference	Pessimistic	Optimistic	Reference	Pessimistic	Optimistic	Reference	Pessimistic	Optimistic	Reference	Pessimistic
AT31	9.3%	8.5%	8.0%	8.8%	11.4%	13.0%	23.3%	24.8%	25.7%	2.5%	3.4%	4.1%	7.0%	7.4%	8.1%
BE21	7.9%	7.2%	0.4%	62.1%	74.3%	113.3%	126.1%	135.9%	140.7%	8.6%	10.6%	16.6%	20.2%	21.8%	22.6%
BE32	11.5%	10.5%	0.7%	41.3%	49.5%	75.0%	92.2%	102.9%	109.1%	6.9%	8.5%	13.2%	18.3%	20.6%	21.8%
BG33	8.8%	2.6%	2.1%	6.4%	7.9%	8.6%	10.2%	10.9%	11.2%	2.8%	3.4%	4.0%	5.1%	5.5%	5.9%
BG42	5.7%	2.0%	0.5%	13.6%	16.6%	17.7%	19.9%	21.3%	21.1%	1.5%	2.5%	3.1%	3.2%	3.8%	4.3%
CZ04	5.8%	5.3%	5.0%	37.0%	41.1%	44.4%	60.8%	65.2%	67.0%	6.2%	7.1%	8.7%	15.2%	16.2%	16.8%
DEA1	0.5%	0.2%	0.1%	34.6%	40.4%	41.8%	47.6%	54.0%	56.5%	8.5%	10.8%	12.0%	15.2%	17.4%	18.2%
DEB3	1.9%	1.8%	1.6%	40.2%	44.4%	46.4%	66.5%	72.1%	69.5%	8.3%	9.4%	10.6%	19.1%	20.7%	21.8%
DEE0	1.9%	1.2%	0.7%	7.6%	8.6%	9.4%	10.6%	12.1%	12.6%	2.6%	3.0%	3.5%	4.4%	5.1%	5.4%
DEF0	0.5%	0.6%	0.5%	12.2%	13.2%	14.2%	39.6%	44.4%	50.7%	2.5%	2.8%	3.1%	9.1%	10.3%	11.8%
EL51	20.6%	18.9%	2.8%	8.1%	9.3%	11.1%	10.3%	10.9%	11.4%	2.3%	2.7%	3.4%	3.0%	3.1%	3.4%
ES24	16.1%	14.7%	5.9%	2.7%	3.3%	4.5%	6.4%	6.9%	7.1%	0.6%	0.7%	1.1%	0.8%	0.8%	0.9%
ES42	10.0%	9.2%	2.3%	2.7%	3.1%	4.4%	4.4%	4.7%	4.8%	0.3%	0.4%	0.7%	0.6%	0.6%	0.7%
ES61	2.6%	2.4%	0.5%	2.6%	3.1%	4.6%	6.5%	7.0%	6.1%	0.3%	0.4%	0.6%	0.9%	1.0%	1.1%
FR10	2.0%	0.3%	0.1%	6.3%	14.7%	16.7%	16.8%	21.2%	25.7%	2.1%	4.8%	5.9%	6.1%	8.1%	10.7%
FRD2	7.0%	1.8%	0.5%	8.7%	13.3%	15.2%	16.8%	18.2%	18.7%	2.7%	4.1%	4.7%	5.3%	5.8%	6.0%
FRF1	13.4%	2.3%	0.5%	14.2%	35.0%	39.8%	43.4%	48.7%	60.9%	2.8%	6.9%	8.0%	8.3%	11.3%	16.6%
HU21	23.4%	21.4%	20.2%	5.6%	6.7%	7.2%	11.2%	12.2%	12.2%	2.3%	2.8%	3.2%	4.8%	5.2%	5.8%
HU31	20.5%	18.8%	17.8%	15.8%	18.4%	19.5%	48.4%	58.3%	50.6%	1.7%	2.4%	3.1%	10.7%	14.8%	15.6%
ITH5	2.1%	0.9%	0.6%	9.1%	10.4%	10.5%	16.4%	18.3%	18.5%	1.5%	2.7%	2.9%	2.9%	6.3%	6.6%

LT02	19.8%	10.6%	4.5%	1.5%	2.8%	3.8%	9.9%	10.7%	11.4%	0.5%	0.8%	1.2%	3.0%	3.3%	3.6%
NL34	26.7%	24.5%	23.1 %	63.9%	73.7%	82.7%	119.0%	126.4%	129.6%	10.3%	12.0%	13.8%	16.9%	17.9%	18.4%
NL42	8.7%	8.0%	7.5%	99.1%	110.9%	117.8%	162.8%	176.6%	187.1%	17.6%	21.4%	24.2%	38.9%	42.4%	45.1%
PL21	2.1%	1.9%	1.8%	21.0%	23.6%	24.2%	39.2%	47.2%	49.1%	3.5%	4.2%	4.7%	9.0%	11.0%	12.4%
PL42	4.3%	4.0%	3.7%	8.2%	9.0%	9.5%	9.1%	9.8%	10.2%	1.8%	2.1%	2.2%	2.0%	2.2%	2.4%
PL52	2.4%	2.2%	2.1%	17.7%	20.4%	21.9%	24.2%	26.8%	27.3%	4.5%	5.3%	6.0%	6.5%	7.8%	8.6%
PL61	3.3%	3.1%	2.9%	7.9%	8.9%	9.5%	12.3%	13.4%	13.5%	2.4%	2.7%	3.0%	4.0%	4.4%	4.6%
PL81	3.3%	3.0%	2.9%	5.9%	6.8%	7.2%	7.3%	7.9%	8.3%	2.0%	2.3%	2.5%	2.6%	2.8%	2.9%
RO12	11.3%	10.3%	8.9%	8.4%	9.4%	9.7%	24.1%	26.0%	26.4%	0.9%	1.2%	1.7%	2.2%	2.3%	2.4%
RO31	5.7%	2.4%	1.4%	2.6%	3.4%	3.7%	4.2%	4.5%	4.6%	1.2%	1.7%	1.9%	2.3%	2.5%	2.6%
SK02	11.3%	4.6%	4.6%	5.7%	8.4%	8.8%	33.7%	36.0%	37.0%	2.5%	3.7%	4.2%	9.4%	10.1%	10.4%
UKC1	4.0%	0.2%	0.0%	64.7%	84.6%	94.1%	72.3%	82.1%	94.1%	8.0%	10.3%	11.7%	8.7%	10.0%	11.7%
UKD6	14.4%	7.3%	0.8%	76.6%	95.6%	106.2%	128.6%	138.1%	141.1%	11.6%	14.2%	16.1%	18.9%	20.7%	21.7%
UKE1	14.1%	0.7%	0.0%	25.4%	36.4%	40.1%	30.7%	35.1%	40.1%	2.6%	3.7%	4.1%	3.1%	3.6%	4.1%

MIN	0.5%	0.2%	0.0%	1.5%	2.8%	3.7%	4.2%	4.5%	4.6%	0.3%	0.4%	0.6%	0.6%	0.6%	0.7%
MEAN	8.9%	6.3%	4.0%	22.0%	27.0%	31.1%	39.8%	43.8%	45.9%	4.0%	5.2%	6.2%	8.5%	9.6%	10.4%
MAX	26.7%	24.5%	23.1 %	99.1%	110.9%	117.8%	162.8%	176.6%	187.1%	17.6%	21.4%	24.2%	38.9%	42.4%	45.1%

Supplementary Table 11 | Feasibility analysis in different cases (optimistic, reference, and pessimistic). The table presents the feasibility under the three criteria identified: (i) impact on grid electricity; (ii) renewable potential, and (iii) land availability at NUTS-2 (Nomenclature of Territorial Units for Statistics) level.

Algorithm	Hyper parameters	CPU times
Heuristic Optimizations		
Nelder-mead	x0 = np.array([2E3,2E3,1E3,1E2,1E4,1E5]); tol = E-15	7min 35s
Nelder-mead - random coordinates	x0 = np.random.randint(1, 1E4, size=(6,)); tol = E-15	7min 33s
L-BFGS-B	x0 = np.array([2E3,2E3,1E3,1E2,1E4,1E5]); tol = E-15	10min 8s
L-BFGS-B - random coordinates	x0 = np.random.randint(1, 1E4, size=(6,)); tol = E-15	1min 36s
Differential Evolution	(pop = 10)	16min 32s
Differential Evolution + Nelder-mead	(pop = 10); tol = E-15	15min 43s + 4min 59s
Mixed Integer Linear Programming		
MILP	n/a	< 1min

Supplementary Table 12 | Optimization models' performance. Tests were conducted using a processor 11th Gen Intel(R) Core (TM) i7-11800H, 16.0 GB RAM. Mixed-Integer Linear Programming (MILP) performed better in all tests conducted. MILP is the option used in the main analysis. L-BFGS-B : Limited-memory Broyden-Fletcher-Goldfarb-Shanno algorithm with Box constraints.

Input parameter	Description	Unit	Value			Note	Source
			Pessimistic	Reference	Optimistic		
Generic input data							
D_{H2}	Hourly hydrogen (H_2) output electrolytic hydrogen production system (EHPS)	kg H_2/h	7,500				4,30,50,51
t	Hours/year	h	8,760				
l	Lifetime EHPS	y	26			26 years lifetime, 25 years operations	
Technology input data							
c_{PV}	Total system cost utility-scale photovoltaics (PV) in euro (EUR) per megawatt (MW)	EUR/MW	1,333,537	537,359	343,032	AT	52
			1,612,776	649,881	414,862	BE	
			1,363,201	549,313	350,663	BG	
			1,612,776	649,881	414,862	CH	
			1,317,754	531,000	338,972	CZ	
			1,291,455	520,402	332,207	DE	
			1,401,138	564,600	360,422	EL	
			1,404,682	566,028	361,333	ES	
			1,739,582	700,979	447,481	FR	
			1,912,240	770,553	491,895	HR	
			1,741,329	701,683	447,930	HU	
			1,441,214	580,749	370,731	IT	
			1,612,776	649,881	414,862	LT	
			1,972,265	794,740	507,335	NL	
			1,612,776	649,881	414,862	NO	
			1,404,845	566,094	361,375	PL	
			1,422,078	573,038	365,808	RO	
			1,612,776	649,881	414,862	SK	
			1,673,080	674,181	430,374	UK	
A_{PV}	Total land area for PV installations in square meters (m^2)/MW	m^2/MW	35,000				53

v_{PV}	Annual Operation & Maintenance (O&M)	EUR/MW	17,273				54
η_{PV}	Efficiency PV system	%	90.0%			Considering inverter, transformers, and rectifier efficiencies	55
c_{WT}	Total system cost utility-scale wind turbines (WT)	EUR/MW	1,421,401	880,278	680,336	AT	52
			1,719,039	1,064,606	822,797	BE	
			1,453,021	899,860	695,470	BG	
			1,719,039	1,064,606	822,797	CH	
			1,404,579	869,860	672,284	CZ	
			1,376,547	852,500	658,867	DE	
			1,493,457	924,903	714,825	EL	
			1,497,234	927,242	716,632	ES	
			1,854,200	1,148,312	887,490	FR	
			2,038,235	1,262,285	975,576	HR	
			1,856,062	1,149,465	888,381	HU	
			1,536,174	951,357	735,270	IT	
			1,719,039	1,064,606	822,797	LT	
			2,102,215	1,301,908	1,006,199	NL	
			1,719,039	1,064,606	822,797	NO	
			1,497,408	927,350	716,716	PL	
			1,515,777	938,725	725,508	RO	
			1,719,039	1,064,606	822,797	SK	
			1,783,316	1,104,413	853,562	UK	
A_{WT}	Total land area for WT installations	m^2/MW	150,000				28
v_{WT}	Annual O&M	EUR/MW	25,455				54
η_{WT}	Efficiency WT system	%	93.0%			Considering transformers, and rectifier efficiencies	55

c_{EL}	Total system cost Alkaline (ALK) electrolyzer	EUR/MW	1,669,387	1,164,612	659,837	First installation in 2024	27
			361,836 in 2031	257,151 in 2033	154,613 in 2034	For the electrolyzer, the 2024 installation also includes project costs, while the following installations refer only to the stack component.	
			329,625 in 2041	215,813 in 2044	107,370 in 2045		
v_{EL}	Annual O&M	% CAPEX	3.0%	2.0%	1.0%		
η_{EL}	Energy demand ALK electrolyzer in kilowatt hours (kWh)/kg H_2	kWh/kg H_2	52.1	50.3	47.3		27
c_{CP}	Unit cost utility-scale H_2 compressors	EUR/kg H_2 compressed per h	9,791			First installation in 2024	30
			8,678 in 2034			Replacements	
			8,122 in 2044				
v_{CP}	Annual O&M	% CAPEX	4.0%				
η_{CP}	Energy demand compressors	kWh/kg H_2 compressed	2.0				30
c_{ST}	Unit cost H_2 storage tanks	EUR/kg H_2 stored	455				56
c_{B}	Unit cost utility-scale Li-ion battery system in EUR/megawatt hour (MWh)	EUR/MWh	423,475	372,444	275,311	First installation in 2024	29,57
			334,652 in 2032	235,638 in 2039	162,536 in 2042	Replacements	
			316,067 in 2040	-	-		
v_{B}	Annual O&M	% CAPEX	2.5%				
η_{B}^{c}	Charging efficiency	%	92.7%			Round trip efficiency 85%	
η_{B}^{d}	Discharging efficiency	%	92.7%				
τ_{B}^{c}	Minimum number time intervals for full charge	n	4				
τ_{B}^{c}	Minimum number time intervals for full discharge	n	4				
λ_{B}	Self-discharge coefficient	% / day	0.2%				
c_{THVLV}	Cost transformer from high voltage (HV) to low voltage (LV)	EUR	33,000,000				58

η_{THVLV}	Efficiency transformer from HV to LV	%	99.0%				55
η_{TLVHV}	Efficiency transformer from LV to HV	%	99.0%				55
c_W	Cost high voltage alternating current (HVAC) wire in EUR/kilometer (km)	EUR/km	1,600,000				58
d	Distance from PV, WT, grid connection and EHPS	km	5				
η_I	Efficiency inverter	%	97.0%				58
Grid input data							
p_E	Price grid electricity for industrial user in EUR/megawatt hour (MWh)	EUR/MWh	122	84	59	AT	59
			205	88	40	BE	
			290	134	62	BG	
			304	123	51	CH	
			108	56	30	CZ	
			220	164	123	DE	
			167	82	41	EL	
			145	62	29	ES	
			276	130	63	FR	
			338	127	48	HR	
			109	52	28	HU	
			351	205	117	IT	
			151	82	46	LT	
			95	63	41	NL	
			126	31	9	NO	
			108	57	31	PL	
			173	99	58	RO	
			211	118	69	SK	
			697	235	82	UK	

γ_E	Carbon intensity grid electricity in kg carbon dioxide equivalent (CO_2e)/MWh	kg CO_2e/MWh	23	23	22	AT	25
			49	48	47	BE	
			108	105	102	BG	
			23	23	23	CH	
			116	113	110	CZ	
			94	91	88	DE	
			135	131	127	EL	
			53	51	50	ES	
			18	17	17	FR	
			46	45	43	HR	
			64	62	60	HU	
			64	63	61	IT	
			49	48	46	LT	
			99	96	94	NL	
			4	4	3	NO	
			211	205	200	PL	
			76	74	72	RO	
			31	30	30	SK	
			101	101	101	UK	
η_E	Transmission losses from grid	%	6%			Considering transformer and rectifier efficiencies	55

Supplementary Table 13 | Input parameters optimization models.

Reference year	2019			2030			2050		
Case	Pessimistic	Reference	Optimistic	Pessimistic	Reference	Optimistic	Pessimistic	Reference	Optimistic
Electrical efficiency (% low heating value)	63%	67%	70%	65%	68%	71%	70%	75%	80%
Unit cost in euro per megawatt (EUR/MW) installed	€ 1,202,543	€ 816,011	€ 429,479	€ 730,115	€ 536,849	€ 343,584	€ 601,271	€ 386,532	€ 171,792
Energy demand in kilowatt hour per kilogram hydrogen (kWh/kg H_2)	53	50	48	51	49	47	48	45	42
Stack lifetime (operating hours)	60000	75000	90000	90000	95000	100000	100000	125000	150000
L (Lifetime in years)	7	9	10	10	11	11	11	14	17

Supplementary Table 14 | Alkaline (ALK) electrolyzer technical and economic characteristics. Authors' elaboration based on data from[27,60,61].

Input parameter	Description	Unit	Electrolyzer technology			Note	Source
			ML	ALK	SOE		
c_{EL}	Total system cost electrolyzer in euro per megawatt (EUR/MW)	EUR/MW	638,339	1,164,612	4,117,381	First installation in 2024	[27,62,63]
			55,544 in 2029	257,151 in 2033	921,429 in 2028	For the electrolyzer, the 2024 installation also includes project costs, while the following installations refer only to the stack component.	
			23,914 in 2034	215,813 in 2044	681,429 in 2034		
			17,704 in 2039	-	502,491 in 2043		
			11,495 in 2044	-	-		
			5,285 in 2049	-	-		
η_{EL}	Energy demand electrolyzer in kilowatt hours per kilogram hydrogen (kWh/kg H_2)	kWh/kg H_2	69.3	50.3	42.2		

Supplementary Table 15 | Electrolyzer technology comparison. Three types of electrolyzer technology are considered: Membraneless electrolyzer (ML), alkaline electrolyzer (ALK), and solid oxide electrolyzer (SOE). Data used in robustness analysis.

Supplementary References

1. European Commission. *EU Delegated Acts on Renewable Hydrogen*. *European Commission* https://ec.europa.eu/commission/presscorner/detail/en/qanda_23_595 (2023).

2. European Commission. *Sustainable Finance Package*. https://finance.ec.europa.eu/publications/sustainable-finance-package_en (2021).

3. Bruninx, K., Moncada, J. A. & Ovaere, M. Electrolytic hydrogen has to show its true colors. *Joule* **6**, 2437–2440 (2022).

4. Egenhofer, C. *et al. For a Study on Composition and Drivers of Energy Prices And Costs in Energy Intensive Industries: The Case of The Chemical Industry - Ammonia*. https://www.ceps.eu/ceps-publications/composition-and-drivers-energy-prices-and-costs-energy-intensive-industries-case/ (2014).

5. Fertilizer Europe. *Map of Major Fertilizer Plants in Europe*. https://www.fertilizerseurope.com/fertilizers-in-europe/map-of-major-fertilizer-plants-in-europe/ (2018).

6. FCHO. *Hydrogen Supply Capacity*. https://observatory.clean-hydrogen.europa.eu/hydrogen-landscape/production-trade-and-cost/hydrogen-production (2022).

7. Nugent, D. & Sovacool, B. K. Assessing the lifecycle greenhouse gas emissions from solar PV and wind energy: A critical meta-survey. *Energy Policy* **65**, 229–244 (2014).

8. Gerloff, N. Comparative Life-Cycle-Assessment analysis of three major water electrolysis technologies while applying various energy scenarios for a greener hydrogen production. *J Energy Storage* **43**, 102759 (2021).

9. Palmer, G., Roberts, A., Hoadley, A., Dargaville, R. & Honnery, D. Life-cycle greenhouse gas emissions and net energy assessment of large-scale hydrogen production via electrolysis and solar PV. *Energy Environ Sci* **14**, 5113–5131 (2021).

10. Petkov, I. & Gabrielli, P. Power-to-hydrogen as seasonal energy storage: an uncertainty analysis for optimal design of low-carbon multi-energy systems. *Appl Energy* **274**, 115197 (2020).

11. Gabrielli, P. *et al.* Seasonal energy storage for zero-emissions multi-energy systems via underground hydrogen storage. *Renewable and Sustainable Energy Reviews* **121**, 109629 (2020).

12. Brownlee, J. *Local Optimization Versus Global Optimization*. https://machinelearningmastery.com/local-optimization-versus-global-optimization/ (2021).

13. Boyd, S. P. & Vandenberghe, Lieven. *Convex Optimization*. (Cambridge University Press, Cambridge, 2004).

14. Kochenderfer, M. J. & Wheeler, T. A. *Algorithms for Optimization*. (MIT Press, Massachusetts, 2019).

15. Brownlee, J. *How to Use Nelder-Mead Optimization in Python*. https://machinelearningmastery.com/how-to-use-nelder-mead-optimization-in-python/ (2021).

16. Nelder, J. A. & Mead, R. A Simplex Method for Function Minimization. *Comput J* **7**, 308–313 (1965).

17. Brownlee, J. *How to Choose an Optimization Algorithm*. https://machinelearningmastery.com/tour-of-optimization-algorithms/ (2020).

18. Georgioudakis, M. & Plevris, V. A comparative study of differential evolution variants in constrained structural optimization. *Front Built Environ* **6**, 102 (2020).

19. Storn, R. & Price, K. Differential evolution - a simple and efficient heuristic for global optimization over continuous spaces. *Journal of Global Optimization* **11**, 341–359 (1997).

20. SciPy. Differential Evolution algorithm. https://docs.scipy.org/doc/scipy/reference/generated/scipy.optimize.differential_evolution.html#r108fc14fa019-5.

21. Mayyas, A., Ruth, M., Pivovar, B., Bender, G. & Wipke, K. *Manufacturing Cost Analysis for Proton Exchange Membrane Water Electrolyzers*. https://www.nrel.gov/docs/fy10osti/72740.pdf. (2019).

22. Morgan, E. R. Techno-Economic Feasibility Study of Ammonia Plants Powered by Offshore Wind. (University of Massachusetts Amherst, 2013). doi:10.7275/11kt-3f59.

23. IEA. *Towards Hydrogen Definitions Based on Their Emissions Intensity*. https://www.iea.org/reports/towards-hydrogen-definitions-based-on-their-emissions-intensity (2023).

24. Ogden, J. M. *Review of Small Stationary Reformers for Hydrogen Production*. https://afdc.energy.gov/files/pdfs/31948.pdf (2002).

25. Bastos, J., Monforti-Ferrario, F. & Melica, G. *GHG Emission Factors for Electricity Consumption. European Commission, Joint Research Centre (JRC)*. http://data.europa.eu/89h/919df040-0252-4e4e-ad82-c054896e1641 (2024).

26. EEA. *Greenhouse Gas Emission Intensity of Electricity Generation in Europe*. https://www.eea.europa.eu/ims/greenhouse-gas-emission-intensity-of-1 (2022).

27. IEA. *The Future of Hydrogen*. https://www.iea.org/reports/the-future-of-hydrogen (2019) doi:10.1787/1e0514c4-en.

28. European Commission. *Preise Landwirtschaftlicher Flächen Nach Regionen*. https://data.europa.eu/data/datasets/wctmbzzccaz8tgh9pqqlg?locale=en (2022).

29. Cole, W., Frazier, A. W. & Augustine, C. *Cost Projections for Utility-Scale Battery Storage: 2021 Update*. https://www.nrel.gov/docs/fy21osti/79236.pdf (2021).

30. Thomas, D., Mertens, D., Meeus, M., Van der Laak, W. & Francois, I. *Power to Gas - Roadmap for Flanders*. https://www.waterstofnet.eu/_asset/_public/powertogas/P2G-Roadmap-for-Flanders.pdf (2016).

31. Parks, G., Boyd, R., Cornish, J. & Remick, R. *Hydrogen Station Compression, Storage, and Dispensing Technical Status and Costs: Systems Integration*. https://www.nrel.gov/docs/fy14osti/58564.pdf (2014) doi:10.13140/RG.2.2.23768.34562.

32. BloombergNEF. *Carbon Offset Prices Could Increase Fifty-Fold by 2050*. https://about.bnef.com/blog/carbon-offset-prices-could-increase-fifty-fold-by-2050/ (2022).

33. *Composition and Drivers of Energy Prices and Costs in Energy Intensive Industries: The Case of the Chemical Industry - Ammonia*. https://aei.pitt.edu/50255/1/CEPS_Energy_Prices_Study_Consolidated_version.pdf (2014).

34. Eurostat. *NUTS - GISCO*. https://ec.europa.eu/eurostat/web/gisco/geodata/reference-data/administrative-units-statistical-units/nuts (2021).

35. FAOSTAT. *Land Use*. https://www.fao.org/faostat/en/#data/RL (2022).

36. Gabrielli, P. *et al.* Net-zero emissions chemical industry in a world of limited resources. *One Earth* **6**, 682–704 (2023).

37. Kakoulaki, G. *et al.* Green hydrogen in Europe – A regional assessment: Substituting existing production with electrolysis powered by renewables. *Energy Convers Manag* **228**, (2021).

38. González-Aparicio, I. *et al.* EMHIRES: European Meteorological-derived HIgh resolution RES dataset. https://zenodo.org/records/8340501 (2021) doi:10.5281/zenodo.8340501.

39. Gonzalez-Aparicio, I. *et al. EMHIRES Dataset Part I: Wind Power Generation*. https://www.researchgate.net/publication/310465577_EMHIRES_dataset_Part_I_Wind_power_generation (2016) doi:10.2790/831549.

40. Gonzalez-Aparicio, I., Huld, T., Careri, F., Monforti, F. & Zucker, A. *EMHIRES Dataset Part II: Solar Power Generation*. https://setis.ec.europa.eu/emhires-dataset-part-ii-solar-power-generation_en (2017) doi:10.2760/044693.

41. UK Government. *UK Low Carbon Hydrogen Standard: Emissions Reporting and Sustainability Criteria*. https://www.gov.uk/government/publications/uk-low-carbon-hydrogen-standard-emissions-reporting-and-sustainability-criteria#:%7E:text=Update%2021%20September%202022&text=The%20standard%20requires%20hydrogen%20producers,to%20the%20’point%20of%20production’ (2023).

42. Sailer, K., Reinholz, T., Lakeit Malin, K. & Crone, K. *Global Harmonisation of Hydrogen Certification.* https://www.weltenergierat.de/wp-content/uploads/2022/01/dena_WEC_Harmonisation-of-Hydrogen-Certification_digital_final.pdf (2022).

43. California Air Resources Board. *CA-GREET3.0 Lookup Table Pathways*. https://ww2.arb.ca.gov/sites/default/files/classic/fuels/lcfs/ca-greet/lut-doc.pdf (2018).

44. US Congress. *Text - H.R.5376 - 117th Congress (2021-2022): Inflation Reduction Act of 2022*. https://www.congress.gov/bill/117th-congress/house-bill/5376/text (2022).

45. Government of Canada. *Chapter 3: A Made-In-Canada Plan: Affordable Energy, Good Jobs, and a Growing Clean Economy*. https://www.budget.canada.ca/2023/report-rapport/chap3-en.html (2023).

46. French Government. *Ordinance No. 2021-167 Relating to Hydrogen*. https://climate-laws.org/document/ordinance-no-2021-167-relating-to-hydrogen_ec1d (2021).

47. Liu, W., Wan, Y., Xiong, Y. & Gao, P. *Green Hydrogen Standard in China: Standard and Evaluation of Low-Carbon Hydrogen, Clean Hydrogen, and Renewable Hydrogen*. https://www.eria.org/uploads/media/Research-Project-Report/RPR-2021-19/15_Chapter-9-Green-Hydrogen-Standard-in-China_Standard-and-Evaluation-of-Low-Carbon-Hydrogen%2C-Clean-Hydrogen%2C-and-Renewable-Hydrogen.pdf (2021).

48. CertifHy. HOME - CERTIFHY. https://www.certifhy.eu/.

49. Green Hydrogen Organisation. *The GH2 Green Hydrogen Standard*. https://gh2.org/our-initiatives/gh2-green-hydrogen-standard (2023).

50. Soloveichik, G. *Future of Ammonia Production: Improvement of Haber-Bosch of Electrochemical Synthesis?* https://ammoniaenergy.org/presentations/future-of-ammonia-production-improvement-of-haber-bosch-process-or-electrochemical-synthesis/ (2017).

51. Saygin, D. *et al.* Ammonia production from clean hydrogen and the implications for global natural gas demand. *Sustainability* **15**, 1623 (2023).

52. IRENA. *Renewable Power Generation Costs in 2020*. https://www.irena.org/publications/2022/Jul/Renewable-Power-Generation-Costs-in-2021 (2021).

53. Denholm, P., Hand, M., Jackson, M. & Ong, S. *Land-Use Requirements of Modern Wind Power Plants in the United States*. https://www.nrel.gov/docs/fy09osti/45834.pdf (2009).

54. Wang, C. *et al.* Optimising renewable generation configurations of off-grid green ammonia production systems considering Haber-Bosch flexibility. *Energy Convers Manag* **280**, 116790 (2023).

55. Tao, M., Azzolini, J. A., Stechel, E. B., Ayers, K. E. & Valdez, T. I. Review—Engineering challenges in green hydrogen production systems. *J Electrochem Soc* **169**, 054503 (2022).

56. Ikäheimo, J., Kiviluoma, J., Weiss, R. & Holttinen, H. Power-to-ammonia in future North European 100 % renewable power and heat system. *Int J Hydrogen Energy* **43**, 17295–17308 (2018).

57. NREL. *Utility-Scale Battery Storage*. *2023* https://atb.nrel.gov/electricity/2023/utility-scale_battery_storage (2023).

58. Salmon, N. & Bañares-Alcántara, R. Impact of grid connectivity on cost and location of green ammonia production: Australia as a case study. *Energy Environ Sci* **14**, 6655–6671 (2021).

59. European Commission. *Dashboard for Energy Prices in the EU and Main Trading Partners*. https://energy.ec.europa.eu/data-and-analysis/energy-prices-and-costs-europe/dashboard-energy-prices-eu-and-main-trading-partners_en (2020).

60. Christensen, A. *Assessment of Hydrogen Production Costs from Electrolysis: United States and Europe*. https://theicct.org/publication/assessment-of-hydrogen-production-costs-from-electrolysis-united-states-and-europe/ (2020).

61. IRENA. *Green Hydrogen Cost Reduction: Scaling Up Electrolysers To Meet The 1.5°C Climate Goal*. https://www.irena.org/-/media/Files/IRENA/Agency/Publication/2020/Dec/IRENA_Green_hydrogen_cost_2020.pdf (2020).

62. Manzotti, A., Robson, M. J. & Ciucci, F. Recent developments in membraneless electrolysis. *Curr Opin Green Sustain Chem* **40**, 100765 (2023).

63. Manzotti, A. *et al.* Membraneless electrolyzers for the production of low-cost, high-purity green hydrogen: A techno-economic analysis. *Energy Convers Manag* **254**, 115156 (2022).
