# Supplementary material for: Effects of emissions caps on the costs and feasibility of low-carbon hydrogen in the European ammonia industry
Source: Nat Commun. 2024 May 4;15:3753. doi: 10.1038/s41467-024-48145-z (PMC11069508; doi:10.1038/s41467-024-48145-z)
Supplement: Supplementary file 3 — Reporting Summary [file 41467_2024_48145_MOESM3_ESM.pdf]

nature portfolio

Corresponding author(s): Stefano Mingolla; Zhongming Lu; Magdalena M. Klemun

Last updated by author(s): Feb 29, 2024

Reporting Summary

Nature Portfolio wishes to improve the reproducibility of the work that we publish. This form provides structure for consistency and transparency in reporting. For further information on Nature Portfolio policies, see our Editorial Policies and the Editorial Policy Checklist.

Statistics

For all statistical analyses, confirm that the following items are present in the figure legend, table legend, main text, or Methods section.

n/a	Confirmed	
☒	☐	The exact sample size (*n*) for each experimental group/condition, given as a discrete number and unit of measurement
☒	☐	A statement on whether measurements were taken from distinct samples or whether the same sample was measured repeatedly
☒	☐	The statistical test(s) used AND whether they are one- or two-sided<br>*Only common tests should be described solely by name; describe more complex techniques in the Methods section.*
☒	☐	A description of all covariates tested
☒	☐	A description of any assumptions or corrections, such as tests of normality and adjustment for multiple comparisons
☒	☐	A full description of the statistical parameters including central tendency (e.g. means) or other basic estimates (e.g. regression coefficient) AND variation (e.g. standard deviation) or associated estimates of uncertainty (e.g. confidence intervals)
☒	☐	For null hypothesis testing, the test statistic (e.g. *F*, *t*, *r*) with confidence intervals, effect sizes, degrees of freedom and *P* value noted<br>*Give P values as exact values whenever suitable.*
☒	☐	For Bayesian analysis, information on the choice of priors and Markov chain Monte Carlo settings
☒	☐	For hierarchical and complex designs, identification of the appropriate level for tests and full reporting of outcomes
☒	☐	Estimates of effect sizes (e.g. Cohen's *d*, Pearson's *r*), indicating how they were calculated

Our web collection on statistics for biologists contains articles on many of the points above.

Software and code

Policy information about availability of computer code

Data collection	No commercial or open source softwares were used to collect data.<br>Data supporting the findings of this study are available within the paper and its supplementary information.
Data analysis	The repository required to replicate the main and sensitivity analyses, complete with all necessary code and input data, is hosted on GitHub and can be accessed at https://zenodo.org/records/10771014. Data analysis was performed using Python 3.9.12, utilizing the following libraries: Pandas, NumPy, Seaborn, SciPy, Matplotlib, and Gurobipy. The optimization model was developed with Gurobipy and solved using the Gurobi solver version 10.0.1. Visualizations were created with Origin2023, Python's Matplotlib and Seaborn libraries, and spatial visualization was conducted with ArcMap 10.7.

For manuscripts utilizing custom algorithms or software that are central to the research but not yet described in published literature, software must be made available to editors and reviewers. We strongly encourage code deposition in a community repository (e.g. GitHub). See the Nature Portfolio guidelines for submitting code & software for further information.

Data

Policy information about availability of data

All manuscripts must include a data availability statement. This statement should provide the following information, where applicable:

- Accession codes, unique identifiers, or web links for publicly available datasets
- A description of any restrictions on data availability
- For clinical datasets or third party data, please ensure that the statement adheres to our policy

Data supporting the findings of this study are available within the paper and its supplementary information. Key data sources include renewable power generation costs from IRENA (https://www.irena.org/publications/2022/Jul/Renewable-Power-Generation-Costs-in-2021), battery system data from NREL ATB (https://atb.nrel.gov/electricity/2023/utility-scale_battery_storage), European ammonia plant locations from Fertilizer Europe (https://www.fertilizerseurope.com/fertilizers-in-europe/map-of-major-fertilizer-plants-in-europe/), SMR hydrogen production sites from FCHO (https://observatory.clean-hydrogen.europa.eu/hydrogen-landscape/production-trade-and-cost/hydrogen-production), geospatial data from EUROSTAT (https://ec.europa.eu/eurostat/web/gisco/geodata/reference-data/administrative-units-statistical-units/nuts), electrolyzer data from IEA (https://www.oecd-ilibrary.org/energy/the-future-of-hydrogen_1e0514c4-en), wind and solar capacity factors from EMHIRES (https://op.europa.eu/en/publication-detail/-/publication/85b2dc7f-aa61-11e6-aab7-01aa75ed71a1/language-en and https://op.europa.eu/en/publication-detail/-/publication/a6c0cf55-45aa-11e7-aea8-01aa75ed71a1/language-en), grid carbon intensity data from JRC-COM-NEEFE (https://data.jrc.ec.europa.eu/dataset/919df040-0252-4e4e-ad82-c054896e1641), and industrial electricity prices from European Commission (https://energy.ec.europa.eu/data-and-analysis/energy-prices-and-costs-europe/dashboard-energy-prices-eu-and-main-trading-partners_en). Data can be requested through the channels provided on each respective website. The processed data supporting the findings of this study are available in the Supplementary Information and at: https://zenodo.org/records/10771014.

Research involving human participants, their data, or biological material

Policy information about studies with human participants or human data. See also policy information about sex, gender (identity/presentation), and sexual orientation and race, ethnicity and racism.

Reporting on sex and gender	n/a
Reporting on race, ethnicity, or other socially relevant groupings	n/a
Population characteristics	n/a
Recruitment	n/a
Ethics oversight	n/a

Note that full information on the approval of the study protocol must also be provided in the manuscript.

Field-specific reporting

Please select the one below that is the best fit for your research. If you are not sure, read the appropriate sections before making your selection.

☐ Life sciences ☐ Behavioural & social sciences ☒ Ecological, evolutionary & environmental sciences

For a reference copy of the document with all sections, see nature.com/documents/nr-reporting-summary-flat.pdf

Ecological, evolutionary & environmental sciences study design

All studies must disclose on these points even when the disclosure is negative.

Study description	This study investigates the effects of emissions caps on the costs and feasibility of low-carbon hydrogen in the European ammonia industry.
Research sample	The study focused on 38 European ammonia plants, selected based on comprehensive data from the Fertilizer Association, the European Commission, and industry producers. These plants are spread across various European regions with differing climates, access to renewable resources, and region-specific cost components. This allows for an assessment of how emission caps impact the costs and feasibility of transitioning to electrolytic hydrogen in ammonia production.
Sampling strategy	No sampling strategy was needed.
Data collection	We integrated multiple source of data to conduct the analysis, including 1) Wind and solar capacity factor at NUTS-2 level in EUROPE: EMHIRES; 2) Renewable power generation cost and performance: IRENA; 3) Electrolyzers cost and performance: IRENA; and 4) Grid costs and emission content: European Commission.<br>S.M. collected the data, which were then validated by A.M., M.J.R., and K.R.<br>All sources and their corresponding links are provided in the reference section and detailed within the Data Availability Statement.

Timing and spatial scale	Our spatial scale analysis is NUTS-2 level in Europe and our time frame ranges from 2024 to 2050.
Data exclusions	The analysis excludes the ammonia plant in Croatia because it lacks capacity factor data from EMHIRES.
Reproducibility	All data used for this study are provided in the main manuscript and supplementary information. We illustrate our analysis step by step for each parameter and result. All calculations and formulations in this study are detailed in the "Methods" section and in supplementary information. Data and calculations needed for our study are published as supplementary files with the article. The source code and data is also available through GitHub for reproducibility (https://zenodo.org/records/10771014).
Randomization	The analysis does not require random sampling and randomization is not involved.
Blinding	Blinding methods are not involved.

Did the study involve field work? ☐ Yes ☒ No

Reporting for specific materials, systems and methods

We require information from authors about some types of materials, experimental systems and methods used in many studies. Here, indicate whether each material, system or method listed is relevant to your study. If you are not sure if a list item applies to your research, read the appropriate section before selecting a response.

Materials & experimental systems

n/a	Involved in the study	
☒	☐	Antibodies
☒	☐	Eukaryotic cell lines
☒	☐	Palaeontology and archaeology
☒	☐	Animals and other organisms
☒	☐	Clinical data
☒	☐	Dual use research of concern
☒	☐	Plants

Methods

n/a	Involved in the study	
☒	☐	ChIP-seq
☒	☐	Flow cytometry
☒	☐	MRI-based neuroimaging

Plants

Seed stocks	n/a
Novel plant genotypes	n/a
Authentication	n/a
